# Optimizing methods for virome analysis based on studies of a synthetic viral community

**DOI:** 10.1128/msystems.00188-26

**Published:** 2026-06-02

**Authors:** Jiayi Duan, Andrew D. Marques, Matthew Hogenauer, Young Hwang, Yanjia Zhang, Aaron Timperman, Stephanie Higgins, Naomi G. Wilson, Elizabeth Aine Fitts, Haeun Karissa Lim, Kyle Bittinger, Ahmed M. Moustafa, Ronald G. Collman, Frederic D. Bushman

**Affiliations:** 1 Department of Microbiology, Perelman School of Medicine, University of Pennsylvania 6572, Philadelphia, Pennsylvania, USA; 2 Department of Bioengineering, School of Engineering and Applied Science, University of Pennsylvania 6572, Philadelphia, Pennsylvania, USA; 3 Department of Biochemistry and Biophysics, Perelman School of Medicine, University of Pennsylvania 6572, Philadelphia, Pennsylvania, USA; 4 Division of Gastroenterology, Hepatology & Nutrition, Children’s Hospital of Philadelphia, Philadelphia, Pennsylvania, USA; 5 Center for Microbial Medicine, Children’s Hospital of Philadelphia, Philadelphia, Pennsylvania, USA; 6 Pulmonary and Critical Care Division, Department of Medicine, University of Pennsylvania Perelman School of Medicine 14640 , Philadelphia, Pennsylvania, USA; The University of British Columbia Okanagan, Kelowna, BC, Canada

**Keywords:** virus, virome, bacteriophage, DNA sequencing, virus enrichment, method optimization

## Abstract

Studies of whole viral populations—the “virome”—are yielding exciting new insights into biological systems, but methods are still being optimized. Here, we describe generation and use of a synthetic viral community and its use to evaluate technical challenges arising in virome analysis. We spiked the mock community into different human sample types, then passed the samples through different virus enrichment protocols and analyzed by Illumina sequencing. Compared with direct metagenomic sequencing, VLP enrichment protocols greatly increased viral read yields from stool and saliva. Four methods for DNA amplification were compared, with three showing over-amplification of small circular ssDNA viruses, most notably GenomiPhi. Studies of viral particle stability in the presence of nuclease showed that most viral genomes were stable when protected in viral particles, but phage MS2 RNA was unexpectedly labile under some of the conditions tested. Comparison of Illumina 1,000-cycle sequencing versus 300-cycle sequencing showed that longer reads supported generation of longer viral genome assemblies. We tested bacteriophage T4 DNA modified with glucosyl-hydroxymethylcytosine (ghmC) and hydroxymethylcytosine (hmC) and found that both were readily detected, though the recovery of ghmC-modified DNA was reduced compared with T4 genomes with unmodified cytosine. These studies together with published data help provide guidance for virome researchers optimizing analytical protocols.

**IMPORTANCE:**

A challenge in characterizing the human virome in health and disease is identifying optimal methods for enriching the viral content of samples. Due to the tremendous abundance and diversity of viruses, capturing as broad of a range of viruses as possible for analysis is difficult and potentially complicated by unrecognized biases. This report presents the use of a synthetic viral community for methods optimization in virome studies and illustrates the feasibility and challenges of current virus enrichment strategies for high-throughput virome analysis of different human sample types.

## INTRODUCTION

The planetary virome is vast. Virus-like particles (VLPs) on Earth are believed to number ~10^31^ (1–4). Rich sea water can harbor ~10^7^ VLPs per mL (5, 6). Human stool can contain ~10^9^ VLPs per gram (7–10). Viruses can affect their hosts in numerous ways, both positively and negatively (9, 11–24). Thus it is often of interest to characterize whole viral populations in a biome to assess the influence of these populations on function.

Due to the vast diversity of viruses, only a small fraction have been characterized, thus many reads from virome sequencing lack close matches in existing databases. The rapid evolution of viral genomes and emergence of variants also make it difficult to know whether sequences in “metagenomic dark matter” are viral in origin (25–27). Moreover, viral sequences often represent only a tiny fraction of nucleic acids in a biological sample, so the overwhelming majority of non-viral nucleic acids complicates the capture and identification of viral sequences. Over 90% of human gut bacterial genomes carry integrated prophages, making it challenging to distinguish free viral particles from integrated sequences (28). Thus, it is often of interest to investigate the virome content in samples after enriching for VLPs before analysis by sequencing. In favorable cases, this will allow tentative identification of previously unstudied sequences as potentially virus-like. Many useful protocols for VLP enrichment have been reported (8, 9, 11, 12, 18, 29–40), but no single method is ideal for all applications.

Greatly complicating virome analysis is the heterogeneity of viral particles. Viral genomes can be comprised of RNA or DNA. Genomes can be single-stranded or double-stranded, linear or circular, and segmented or continuous. Viral particles can be of many morphologies, and enclosed in one or more lipid membranes. Viral particles can range in size from 1.5 μm in length (pandoravirus) to 25 nm (Adeno-associated virus). Genome sizes range from 2.8 Mb for Pandoravirus to 2–3.9 Kb for Anelloviruses (4, 41, 42). Genomes of satellite viruses and virus-like RNAs can be even smaller, comprised of structured circular molecules as little as 200–400 nt in length (27, 43, 44).

Here, we describe steps toward optimizing methods for VLP enrichment based on assays with a synthetic viral community comprised of known viruses. Several previous publications have also investigated virome methods with spike-ins of known viruses (18, 31, 33–36, 39, 45–48), which complement the work described here. As new virome research programs are initiated, interest turns to capturing viruses that might have previously been lost during purification, motivating further rounds of method optimization. We included eight viruses in our mock community, named VirMock1, spanning a wide range of viral types (Table 1). Several questions pertinent to optimizing methods were addressed. Multiple methods for VLP enrichment have been reported (e.g., 8, 18, 29, 32, 34, 37, 38, 40, 45–47, 49)—how do these perform versus simple metagenomic DNA sequencing or RNA-seq on the same samples? When only small amounts of enriched viral genomes are available, amplification methods are often used—how much do these distort the relative abundances of viral genomes in a sample? How does the choice of sequencing method affect the data recovered? VLP fractions are often treated with nucleases (50) to remove host cell nucleic acids—how frequently do these treatments also destroy viral genomes? Bacteriophage DNA has been reported to be modified by at least 10 forms of covalent modification (51–53)—how often do these disrupt recovery in typical virome protocols? A complication is that many factors can influence the answers to these questions. Here, we start with several published protocols and report surveys using our synthetic viral community to address some of the parameters influencing the output virome data produced. Examples of optimized protocols are presented in Document S1.

**TABLE 1:** Viruses included in the synthetic viral community VirMock1

Virus	Enveloped?	RNA or DNA?	Single-stranded (ss) or double-stranded (ds)?	Morphology	Genome size (bp)	Host
Enterobacteria phage T4	No	DNA	ds	Tailed phage	168,903	*Escherichia coli* DH10B
Enterobacteria phage lambda	No	DNA	ds	Tailed phage	48,502	*Escherichia coli* DH10B
Adeno-associated virus vector (AAV)	No	DNA	ss	Icosahedral	3,300	Synthetic vector grown in human cell line
Vaccinia virus WR (VV)	Yes	DNA	ds	Brick-shaped	194,711	BSC-1, African green monkey kidney cells
Pseudomonas phage phi6	Yes	RNA	ds	Spherical	13,385 (all segments total)	*Psuedomonas syringae* LM2691
Phage MS2	No	RNA	ss	Icosahedral	3,569	*Escherichia coli* C3000
Murine hepatitis virus strain A59 (MHV)	Yes	RNA	ss	Spherical	31,335	17CI-1, murine fibroblast cell line
Enterobacteria phage M13	No	DNA	ss	Filamentous	6,407	*Escherichia coli* C3000

## RESULTS

### Comparison of methods for recovering VLP contigs

**Fig 1:**
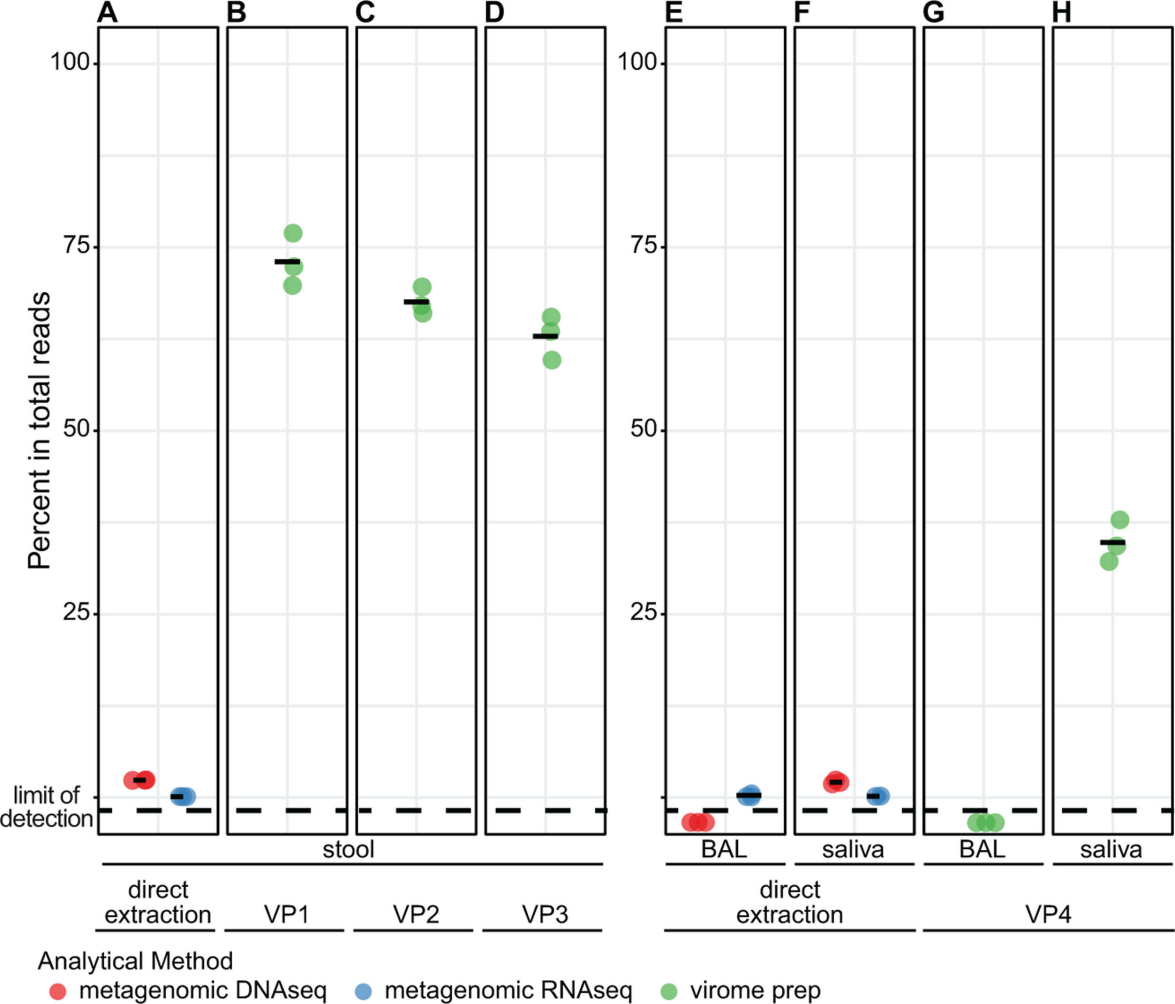
Comparison of viral sequence recovery after virus particle enrichment followed by sequencing, versus direct metagenomic sequencing of total DNA or RNA. (**A–D**) Percentages of reads mapped to viruses annotated by Cenote-Taker2 in stool that was analyzed by (A) direct extraction of DNA (red) or RNA (blue), or after viral particle enrichment using the (**B**) VP1, (**C**) VP2, or (**D**) VP3 protocols (green), with the mean of triplicate measurements represented by a black bar. (**E–H**) Percentages of reads mapped to viruses annotated by Cenote-Taker2 in (**E and G**) BAL or (**F and H**) saliva that was analyzed by (**E and F**) direct extraction and metagenomic DNAseq (red) and RNAseq (blue), or after (**G and H**) viral particle enrichment using the VP4 protocol (green), with the mean of triplicate measurements represented by a black bar. Dots below the limit of detection represent samples, with no viral contigs detected.

We initially compared viral sequence recovery after VLP enrichment versus simple metagenomic DNA or RNA sequencing of the unfractionated samples. For this, we first monitored just the viruses naturally occurring in the stool community analyzed. Three methods were compared for VLP enrichment from stool samples (Table 2), using methods derived from published protocols and used in multiple studies, here termed VP1 (46), VP2 (47), and VP3 (45). All methods involved homogenization of stool in SM buffer, centrifugation to remove debris, filtration, nuclease treatment, and nucleic acid extraction. The methods differ in the details, including amount of starting material used, extent of initial dilution, filtration method, whether samples were concentrated after filtration, nature of the nuclease treatment, whether proteinase K treatment and phenol extraction were performed, and the nucleic acid purification kit chosen. Extracted RNA was subjected to cDNA synthesis and DNA and cDNA pooled and sequenced together. All three methods were tested on a common sample of homogenized stool (Fig. 1A through D). For comparison, we also analyzed viral sequence recovery from the same stool samples in pipelines based on conventional metagenomic DNA sequencing and total RNA-seq. Output sequencing reads were analyzed using Sunbeam (54), which includes Cenote-Taker2 for viral content annotation (55), then compared over the percentage of reads mapping to the viral contigs generated from the data.

**TABLE 2:** Steps in each of the three virus enrichment protocols focused on analysis of stool samples

Steps	VP1	VP2	VP3
Homogenization	200 mg stool resuspended in 10 mL SM buffer	50 mg stool resuspended in 500 μL SM buffer	500 mg stool resuspended in 10 mL SM buffer
Centrifugation	4,000 × *g* for 10 min	17,000 × *g* for 3 min	5,000 × *g* for 10 min (twice)
Filtration	0.22-μm Steriflip vacuum filter	40-μm pore-size strainer; then 0.8-μm PES centrifugal filter, 17,000 × *g* for 1 min	0.45-μm PES syringe filter (twice);
			0.5M NaCl and 10% PEG-8000. Incubate overnight (16 h) at 4°C;
			5,000 × *g* for 20 min to collect pellet;
			Pellet suspended in 400 μL SM buffer + 400 μL chloroform to extract;
			2,500 × *g* for 5 min
Reconcentration	Amicon Ultra-15 centrifugal filter, 4,000 × *g* for 10min intervals till ~ 250 μL left;		
	Add 10 mL SM buffer and repeat the previous centrifugation step		
Nuclease treatment	10× DNase buffer + 2 μL DNase I + 1 μL RNase I (Roche) for every 100 μL of post-viromeprep sample, 37°C incubation for 30 min		Aspirate the aqueous phase and mix it with 10 mM CaCl_2_, 50 mM MgCl_2_, 8 U TURBO DNase I and 20 U RNase I, 37°C incubation for 1 h;
	Add 1 μL of 0.5 M EDTA and incubate at 75°C for 10 min		Incubate at 70°C for 10 min
Proteinase K treatment			Proteinase K and 10% SDS for 20 min at 56°C
Nucleic acid extraction	QIAamp Viral RNA mini kit		100 μL phage lysis buffer. Incubate at 65°C for 10 min;
			Phenol/chloroform/isoamyl alcohol extraction (twice);
			Centrifuge at 8,000 × *g* for 5 min;
			DNeasy blood and tissue purification kit

Results with VP1–VP3 showed recovery of viral contig-aligning reads (DNA and cDNA combined) reaching an average of 62.9 (±2.98)% to 73.0 (±3.60)% of total reads, while metagenomic DNA- and RNA-seq returned only an average of 2.37 (±0.04)% and 0.128 (±0.011)%, respectively (Fig. 1A through D). The majority of recovered contigs annotated as Caudoviricetes, tailed dsDNA bacteriophages, in both virome sequencing and metagenomic sequencing (Fig. S1B and G through I). Metagenomic sequencing was less efficient at recovering viral genomes, due in part to dilution with DNA from other sources. Thus, we conclude that VP1–VP3 are all effective at enriching for virus-like particles and increasing recovery of virus-like contigs from stool.

We next carried out a similar comparison for saliva and BAL (Fig. 1E through H). The two represent sample types with lower biomasses than stool, though saliva is still rich in viruses. BAL of healthy individuals, in contrast, is quite sparse for viruses, cells and biological materials generally, thus yielding a low amount of DNA for sequencing (49, 56–59). For analysis of these liquid samples, an enrichment protocol named VP4 was devised, paralleling VP1 but with reduced sample dilution and use of filters with larger pore sizes, which was added in an attempt to accommodate the higher viscosity of some airway samples.

Saliva undergoing VP4 had on average 34.8 (±2.86)% of reads annotated as viral (Fig. 1H). Saliva analyzed using metagenomic DNAseq and RNAseq without enrichment yielded only 2.10 (±0.26)% and 0.179 (±0.026)% viral reads, respectively (Fig. 1F). For BAL, VP4 resulted in no recovery of viral contigs (Fig. 1G); repeating the bioinformatic analysis using geNomad (60) for viral annotation returned similar result (Fig. S2). Processing of samples with low biomass through the VLP enrichment protocols, including BAL, OP wash and nasopharyngeal swab eluates, commonly resulted in sample loss. This indicates that other analytical approaches may be needed for low-biomass samples (discussed below) (49, 56–59).

To ask how these methods perform with viruses of widely differing particle compositions, we next turned to tests of known viruses spiked into human-derived samples.

### Assembling the VirMock1 synthetic viral community

Viral particles vary extensively in genome sizes, structures, and nucleic acid types. As a tool for methods optimization, we thus assembled a community with a wide range of virus types (VirMock1), where each virus was well characterized and straightforward to grow (Table 1). We included viruses of multiple morphologies with RNA genomes (phi6, MS2, and MHV) and DNA genomes (AAV, lambda, M13, T4, and VV), including both single-stranded and double-stranded representatives.

To analyze an example of unusual covalent DNA modification, we included phage T4 and two mutant derivatives (Table S1) and assessed the effects of the DNA modification of each strain on virome recovery (see below). In our standard VirMock1 community, we used wild-type T4 only.

We acknowledge that different viruses behave differently, and the applicability of the study result is limited to the viruses in VirMock1. In future studies, it will be beneficial to incorporate more varieties of viruses, such as giant viruses, to provide more comprehensive insights into virome analysis.

### Use of VirMock1 to test protocols for virus recovery from stool

**Fig 2:**
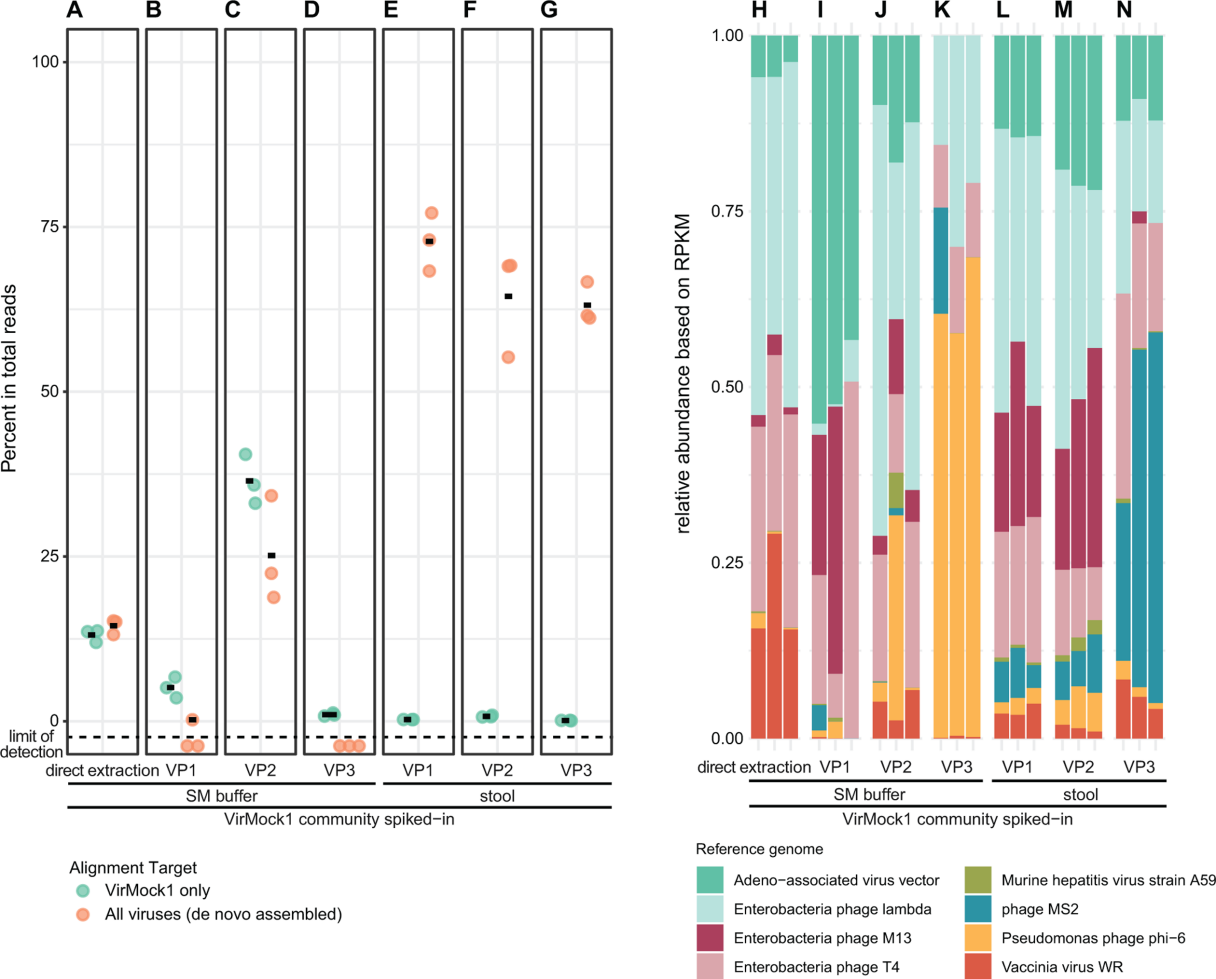
Comparison of methods for purifying viral particles from stool. (**A–G**) Percentage of reads mapping to viruses from VirMock1 or any *de novo* assembled viral contigs after VirMock1 was spiked into (**A–D**) SM buffer or (**E–G**) stool. Samples were (**A**) nuclease treated and then directly extracted for nucleic acid, or first enriched for viral particles using (**B and E**) VP1, (**C and F**) VP2, or (**D and G**) VP3. Reads aligning to any virus as annotated by Cenote-Taker2 are indicated in tan; reads annotating as aligning to a virus in VirMock1 are shown in green; the mean of triplicate measurements is represented by the black bar. Dots below the limit of detection represent samples with no viral contigs detected. (**H–N**) Relative abundance of each VirMock1 reference virus in samples where VirMock1 was spiked into (**H–K**) SM buffer or (**L–N**) stool. Samples were (H) nuclease treated and then directly extracted for nucleic acid, or first enriched for viral particles using** (I and L**) VP1, (**J and M**) VP2, or (**K and N**) VP3.

To test virome recovery using VP1–VP3, we spiked the VirMock1 community into a common stool sample or into SM buffer. Purified virome nucleic acids were analyzed using Illumina short-read sequencing. Sequence reads were (i) directly mapped to genomes of VirMock1 community members to quantify VirMock1 recovery and (ii) used to generate contigs, followed by contig annotation, and then mapping of reads back onto viral contigs to quantify all viruses in the samples, not limited to VirMock1. Each sample type was analyzed in triplicate (Fig. 2). Fig. 2A through G quantifies the relative proportions of reads of VirMock1 and total viruses annotated; Fig. 2H through N shows the relative proportions of the VirMock1 community members recovered. As a check, unspiked biological samples were analyzed for representation of the VirMock1 viruses, and no significant matches were detected (Table S2).

As a reference, the VirMock1 community was mixed with SM buffer, treated with nuclease (DNase I and bovine pancreatic RNase, Methods S1), and directly extracted (Fig. 2A and H). After direct extraction, an average of 13.1 (±0.976)% of reads aligned to the VirMock1 genome sequences (Fig. 2A, green dots). Assembly of all viral contigs and mapping of reads back to contigs yielded a similar result (Fig. 2A, tan dots).

A similar analysis was performed after carrying out the VP1-VP3 purification protocols on VirMock1 spiked into a common stool sample (Fig. 2E through G), or spiked into SM buffer (Fig. 2B through D). After spiking into the stool sample, for VP1, viral contigs aligned to an average of 72.8 (±4.41)% of reads (Fig. 2E, tan dots); numbers were slightly lower for VP2 (64.5% ± 8.00%) (Fig. 2F, tan dots) and VP3 (63.1% ± 3.06%) (Fig. 2G, tan dots). The proportions of the VirMock1 sequences in these samples were much lower, in the range of 0.1%–0.8% of all reads, but this reflects dilution by the large numbers of viral sequences authentically present in the stool samples and recovered after viral enrichment.

In contrast, the recovery of VirMock1 sequences was more variable after spiking into SM buffer only (Fig. 2B through D). VP2, the method involving the least dilution of the sample, showed the best recovery, while heavy losses of VirMock1 were seen with VP1 and VP3. This comparison is consistent with the idea that dilution can impair recovery, and that biological materials such as stool may improve recovery, potentially via “carrier” effects.

Fig. 2H through N shows the VirMock1 lineages detected in each sample as read out from the Illumina sequencing data. Direct extraction of VirMock1 treated only with nuclease before purification showed robust representation of six of the VirMock1 viruses, but two RNA viruses, phage MS2 and murine hepatitis virus, were present in only trace amounts. All eight VirMock1 viruses were detected after spiking into stool, again suggesting a possible “carrier” effect of the biological sample, in which the presence of stool components may stabilize labile viruses and facilitate their recovery. Potential carrier effects were also seen with saliva, where recovery was better than for BAL, oropharyngeal (OP) wash or SM buffer (Fig. S3A through E). Principal coordinate analysis (PCoA) indicated that experimental treatments affected the viruses recovered: sequencing results clustered more closely when spiked into stool than spiked into SM buffer, again consistent with the potential carrier effect of stool components (Fig. S4).

Fig. S5 shows the annotation of all viral contigs—mostly stool derived—in each specimen. The samples of stool with VirMock1 spiked in were dominated by Caudoviricetes (Fig. S5A through C), an abundant lineage of tailed bacteriophages with double-stranded DNA genomes, and Malgrandaviricetes, a group containing common single-stranded DNA bacteriophages, both widely reported in human stool (13, 24, 37, 61–67).

The nature of background contamination in VirMock1 could also be assessed in the sequence data. Analyzing VirMock1-spiked SM buffer with Kraken2 showed that the majority of the reads not assigned to VirMock1 viruses were from bacterial and mammalian host cells used to propagate the viruses (Fig. S6A and H). Identification and removal of background sequences are important steps in virome analysis (68–71).

### Distortions of recovered communities associated with different DNA amplification and library preparation methods

We next tested how different DNA amplification methods affected virome recovery. Since stool is rich in viruses and may not require amplification, we focused instead on liquid samples undergoing VP4. We assessed VirMock1 (i) spiked into saliva and purified via VP4, (ii) spiked into SM buffer and purified via VP4, or (iii) spiked into SM buffer, nuclease-treated, and directly extracted. Samples were then reverse-transcribed and amplified with one of four commercially available amplification kits: GenomiPhiV3 (72), primary template-directed amplification (PTA) (73), whole transcriptome amplification (WTA2) (74), or multiple annealing and looping-based amplification cycles (MALBACs) (75) prior to Illumina DNA sequencing. These include multiple displacement amplication and quasi-linear amplification, with detailed features of each method specified in Table S3. We also included analysis of VirMock1 with no amplification. Samples were analyzed in triplicate.

**Fig 3:**
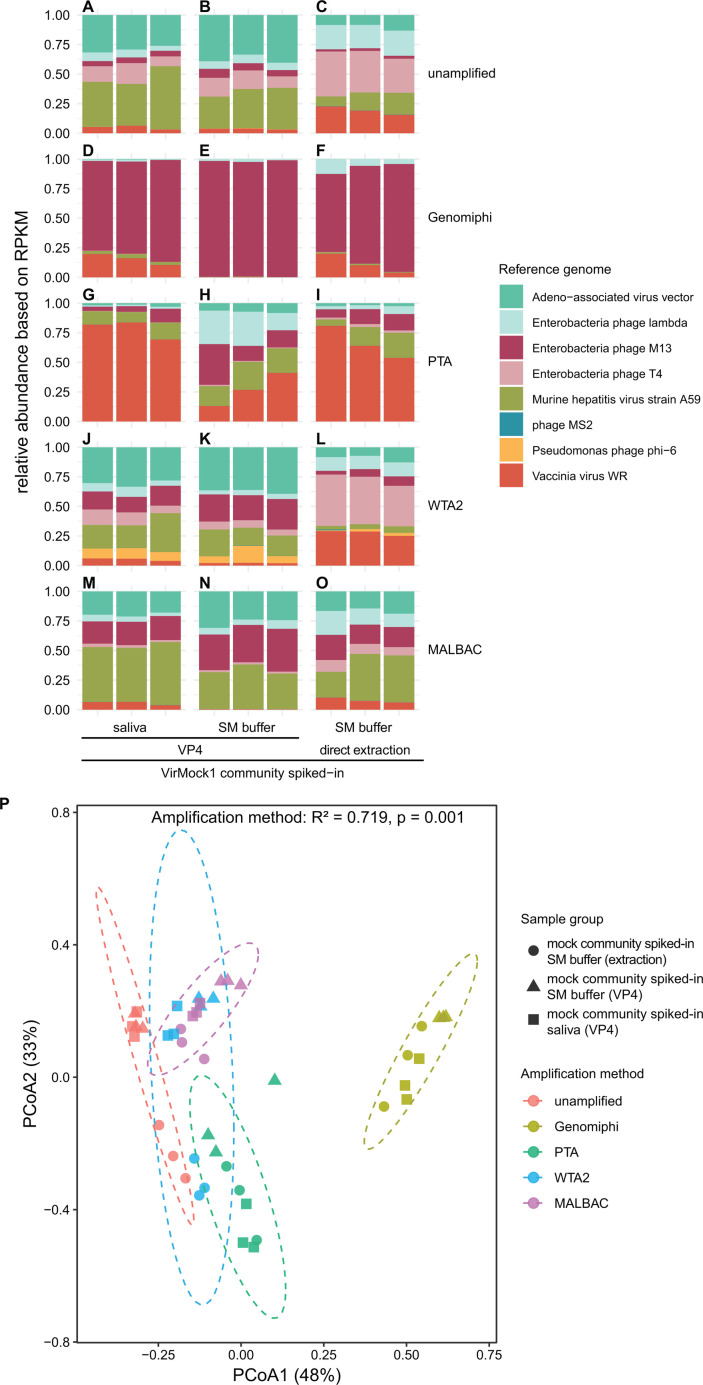
Comparison of the effects of different DNA amplification methods on viral genome recovery. (**A–O**) Relative abundance of each VirMock1 reference virus. Samples were compared after addition of VirMock1 to (**A, D, G, J, and M**) saliva or (**B, E, H, K, and N**) SM buffer and purified using a modified protocol with elements of VP1 and VP2 optimized for saliva (VP4), or (**C, F, I, L, O**) after addition of VirMock1 into SM buffer, nuclease treated and extracted immediately afterwards. Samples were analyzed by Illumina sequencing without amplification, or after amplification using (**D–F**) GenomiPhi, (**G–I**) PTA,( **J–L**) WTA2 with 17 PCR cycles, or (**M–O**) MALBAC with 17 PCR cycles. (**P**) PCoA was performed using Bray-Curtis distances computed based on relative abundance of VirMock1 reference virus in each sample. Variation in community composition across different amplification methods were assessed using permutational multivariate analysis of variance (PERMANOVA) on the Bray-Curtis distances (*R*^2^ = 0.719, *P* = 0.001). Ellipses showing the 95% CI around the group mean were drawn for sample groups with more than three samples, assuming a multivariate t-distribution. Amplification methods (represented by different colors) and samples (represented by different shapes) were as in panels A–O.

In the VirMock1-spiked SM buffer samples treated with nuclease and directly extracted without amplification (Fig. 3C), the sequencing result generally reflected the input VirMock1 composition (Table S4). Samples showed good recovery of six of the VirMock1 viruses, and trace amount of phi6 and MS2. Roughly similar patterns were seen for the VP4-purified unamplified samples (Fig. 3A through C), except in these samples MHV and AAV showed increased representation after purification.

Different amplification methods resulted in different biases for the VirMock1 community members. GenomiPhi greatly favored M13 (Fig. 3D through F) probably reflecting rolling circle replication of the small circular ssDNA genome, as has been reported in several previous studies (33, 76, 77). The other three methods showed less extreme distortions of the viral populations (Fig. 3G through O). PTA did not show such overamplification of M13 but resulted in considerable amplification of vaccinia virus (Fig. 3G through I). A repeat experiment with PTA showed less positive bias for vaccinia, while M13 was over-amplified even more dramatically (Fig. S7E through H). WTA2 and MALBAC also showed lower and variable levels of overampification of M13 in some conditions (Fig. 3J through O). Amplification using MALBAC showed overall similar relative abundance of VirMock1 viruses compared with unamplified, except for a moderate bias favoring MHV and M13 (Fig. 3M through O).

Fig. 3P summarizes these data by PCoA based on Bray-Curtis distances between the measured VirMock1 compositions of samples. Samples clustered by amplification method (Fig. 3P; PERMANOVA, Bray-Curtis, *R*^2^ = 0.719, *P* = 0.001). Genomiphi was the most extreme outlier, with the other methods closer to unamplified but clustering separately. Thus we conclude that the GenomiPhi method is too biased for routine use, and the other methods recovered most viruses effectively, though each has its own modest biases; it will be useful to characterize further amplification methods over additional sample types in future work.

We also compared methods for second-strand cDNA synthesis after reverse transcription (RT; Fig. S8). Viral genome recovery was actually efficient without implementing any specific step for second strand synthesis after RT using our custom protocol (Fig. S8A and H), likely due to the spontaneous second-strand synthesis initiated by RT under the conditions used. However, addition of Klenow polymerase in a subsequent step appeared to diminish the variability in viral proportions (Fig. S8O; linear regression, *P* < 2e−16). Thus, in our favored protocol (Methods S1), we recommend a second-strand synthesis step.

### Virus types differ in their sensitivity to nuclease treatments

**Fig 4:**
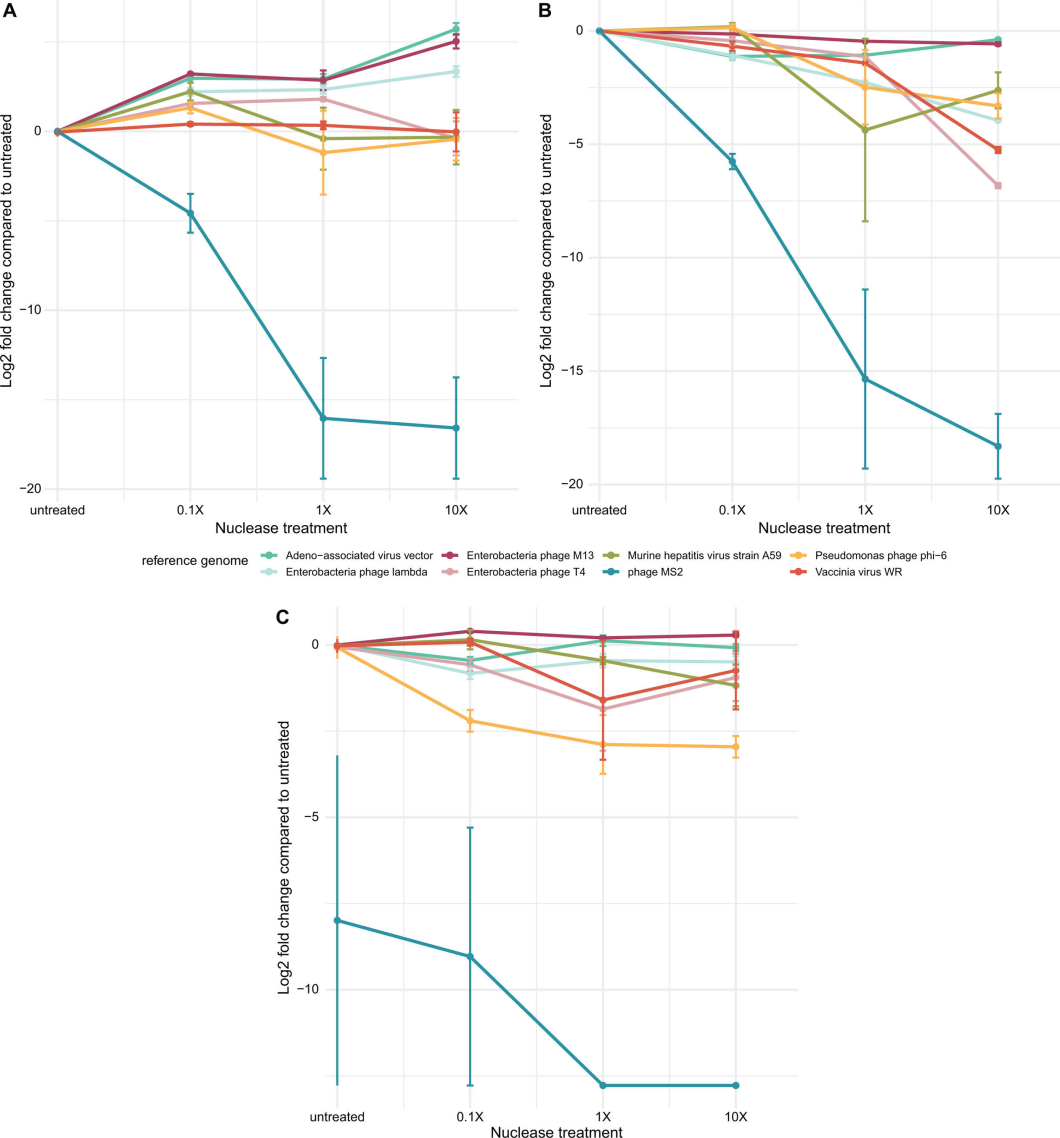
Assessing the stability of viral genomes within viral particles in the presence of nucleases. Nuclease treatment titrated from no added nuclease to 10 times of 1× nuclease was performed in the presence of VirMock1. 1× nuclease included 10× DNase buffer, 2 μL of Roche DNase I, and 1 μL of bovine pancreatic RNase for every 100 μL of post-virome prep sample (Methods S1). Dots represent the mean log_2_ fold changes of relative abundance or genome copy number of a reference virus when compared with the mean relative abundance or genome copy number of that virus in the untreated control triplicates, with lines connecting the mean log_2_ fold changes across nuclease concentrations and error bars representing the standard errors of the mean log_2_ fold change. Spearman’s rank correlations were computed between the log_2_ fold changes of relative abundance and nuclease concentration for each virus to summarize the overall trend with increasing nuclease treatment. (**A**) Log_2_ fold change of relative abundance of each virus (calculated based on RPKM) after VirMock1 underwent different nuclease treatments, followed by Illumina sequencing. Spearman’s rank correlation, AAV: ρ = 0.907, *P* = 4.7e−5; M13: ρ = 0.842, *P* = 5.9e−4; lambda: ρ = 0.928, *P* = 1.3e−5; VV: ρ = 0.367, *P* = 0.24; MHV: ρ = −0.108, *P* = 0.74; phi6: ρ = −0.0864, *P* = 0.79; T4: ρ = 0, *P* = 1; MS2: ρ = −0.901, *P* = 6.3e-5. (**B**) Log_2_ fold change of genome copy number of each virus after VirMock1 underwent different nuclease treatments, followed by qPCR. Spearman’s rank correlation, AAV: ρ = −0.324, *P* = 0.3; M13: ρ = −0.885, *P* = 1.3e−4; lambda: ρ = −0.972, *P* = 1.4e−7; VV: ρ = −0.972, *P* = 1.4e−7; MHV: ρ = −0.777, *P* = 2.9e−3; phi6: ρ = −0.756, *P* = 4.5e−3; T4: ρ = −0.95, *P* = 2.3e−6; MS2: ρ = −0.907, *P* = 4.7e−5. (**C**) Log_2_ fold change of relative abundance of each virus (calculated based on RPKM) after VirMock1 was spiked into stool and underwent VP1 with different nuclease treatments, followed by Illumina sequencing. Spearman’s rank correlation, AAV: ρ = 0.043, *P* = 0.89; M13: ρ = 0.389, *P* = 0.21; lambda: ρ = −0.345, *P* = 0.27; VV: ρ = 0.173, *P* = 0.59; MHV: ρ = −0.734, *P* = 6.6e−3; phi6: ρ = −0.734, *P* = 6.6e−3; T4: ρ = −0.367, *P* = 0.24; MS2: ρ = −0.415, *P* = 0.18.

VLP enrichment methods typically involve a nuclease step following VLP enrichment and before nucleic acid purification to remove free nucleic acids (8, 16, 18, 19, 29, 31–34, 36–39, 48, 61, 62, 76, 78–80). However, such a step has the danger of degrading nucleic acid within particles if there is any degree of accessibility of encapsulated viral genomes (8, 78, 79, 81). We thus incubated VirMock1 in SM buffer with a titrated mixture of DNase and RNase over a range of concentrations, and quantified viral genome recovery. Quantification was carried out using Illumina DNA sequencing (Fig. 4A) or qPCR (Fig. 4B). We also compared results after spiking VirMock1 into stool (Fig. 4C). Recovery of each virus in VirMock1 was quantified relative to the measured input as represented by the untreated condition.

**Fig 5:**
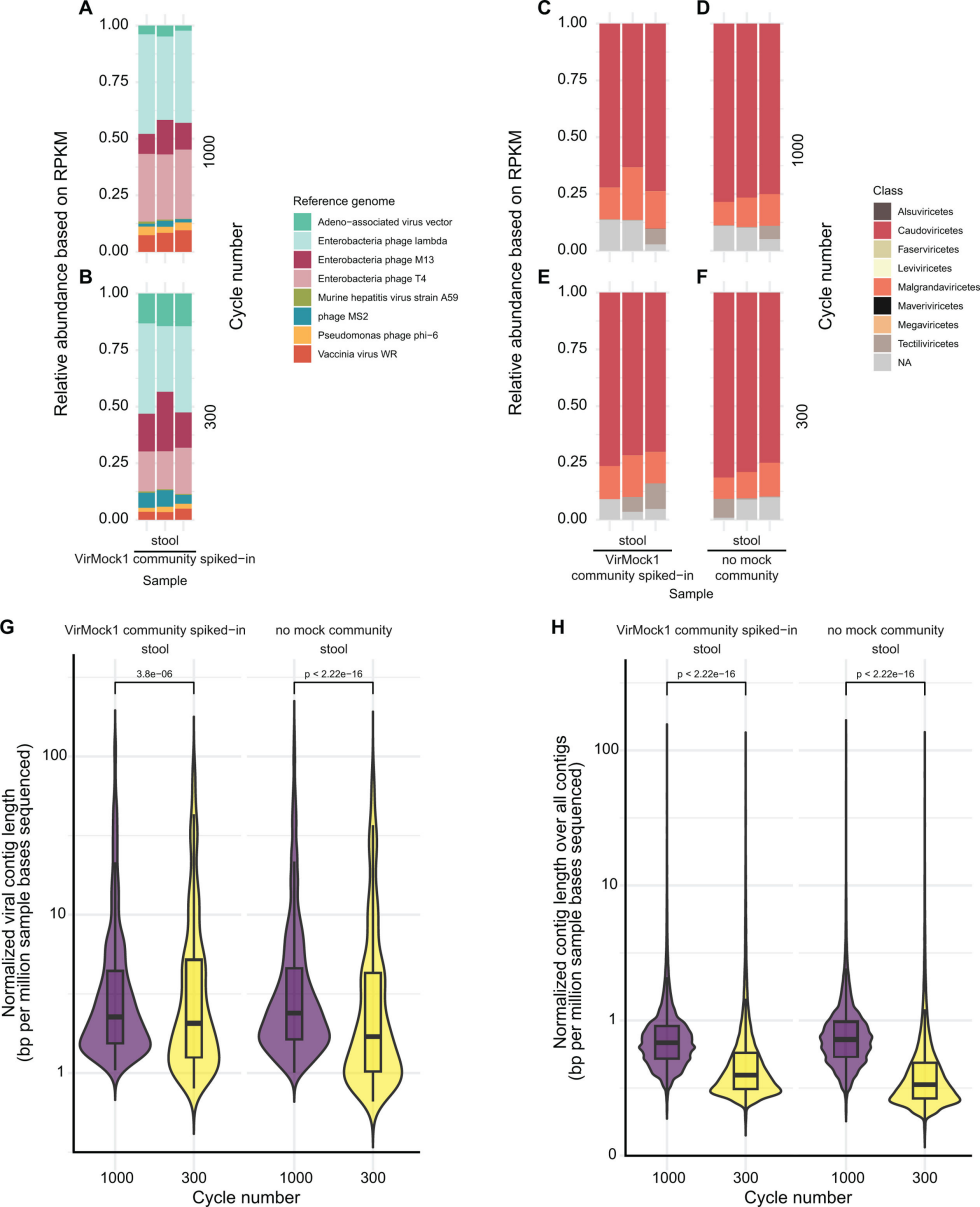
Comparison of Illumina sequencing with the Illumina 1,000-cycle kit and 300-cycle kit platforms. Stool was unspiked or spiked with VirMock1 community, underwent VP1 and Nextera XT library preparation, and sequenced using either the 300-cycle kit or 1,000-cycle kit. (**A and B**) Relative abundance of each reference virus in VirMock1-spiked stool when sequenced using (**A**) the 1,000-cycle kit or (**B**) the 300-cycle kit. (**C–F**) Relative abundance of classes of viruses as annotated by Cenote-Taker2 in (**C**) VirMock1-spiked stool when sequenced using the 1000-cycle kit or (**E**) the 300-cycle kit, and (**D**) unspiked stool when sequenced using the 1,000-cycle kit or (**F**) the 300-cycle kit. (**G**) Distribution of the normalized length of the viral contigs output by the 1,000-cycle kit and 300-cycle kit in VirMock1 community-spiked and unspiked stool. (**H**) Distribution of the normalized length of all contigs (not only viral) output by the 1,000-cycle kit and 300-cycle kit in VirMock1 community-spiked and unspiked stool. Contig lengths were normalized to total sample sequencing depth (as expressed by base pairs per million sample bases sequenced) and plotted on a log_10_ scale. Violin plots show the full distribution of normalized contig lengths, with embedded boxplots indicating the median and interquartile range. Pairwise differences between the kits were evaluated using Wilcoxon rank-sum tests, with formatted *P*-values shown above each comparison. Purple: 1,000-cycle kit. Yellow: 300-cycle kit.

Recovery of viruses varied among types. Most viruses were insensitive to nuclease up to 10 times (10×) the concentration used in typical VLP preparations (Fig. 4A and B). An exception was bacteriophage MS2, which was sharply reduced in abundance by both measures at even 0.1× nuclease concentration (Fig. 4A and B). The decreased MS2 was associated with a proportional increase of other viruses in the sequencing data (Fig. 4A). MS2 was also lost when spiked into stool in two out of three replicates even without nuclease treatment, perhaps due to handling or pre-existing nuclease activity in stool (Fig. 4C). In other cases where MS2 was spiked into stool (Fig. 2 and 5 below), MS2 genomes were detected, but in reduced proportion relative to other VirMock1 viruses. Other viruses showed less susceptibility to nuclease; phi6 showed some decrease in relative abundance when spiked into stool (Fig. 4C). Control experiments showed that free nucleic acids were reduced in abundance efficiently by 1× nuclease treatment when passed through VP4 (Table S5). Thus, we find that most viruses withstood the nuclease treatment robustly, but one non-enveloped linear RNA virus (MS2) was quite sensitive.

### Comparing Illumina short-read versus longer read sequencing

To begin to examine how the choice of sequencing method affects the output contig length, we compared the new Illumina 1,000-cycle kit and the standard 300-cycle kit. For this, we used the same sequencing libraries generated from a common stool sample spiked with VirMock1 and purified using VP1 (Fig. 2L and 5). Samples were sequenced and analyzed using our contig assembly and annotation pipeline (Methods S1).

The proportions of virus types in the stool samples were similar between the 1,000-cycle kit and 300-cycle kits for both the VirMock1-spiked (Fig. 5A, B, C, and E) and unspiked samples (Fig. 5D and F). Mock community members were detected after spike-in of VirMock1 (Fig. 5A and B), and an unbiased virus annotation emphasized Caudoviricetes and Malgrandaviricetes, as expected for stool samples (Fig. 5C through F).

We next compared the distribution of contig lengths. For comparison, we normalized the length of each contig by the total number of bases sequenced in that sample. The 1,000-cycle kit resulted in more total contigs and viral contigs of longer length than the 300-cycle kit for both the VirMock1-spiked and unspiked stool samples (Fig. 5G and H). The median length of contigs assembled from spiked and unspiked stool was 576 and 587 bp, respectively, using the 1,000-cycle kit, and 449 and 450bp, respectively, using the 300-cycle kit (Table S6). Thus, use of the longer length Illumina sequencing platform resulted in longer length contigs (Wilcoxon rank-sum, *P* < 0.001). As expected, different sequencing depths also resulted in different length contigs (linear regression, *R*^2^ = 0.338, *P* = 1.8e−16; Table S7; Fig. S9).

### Beginning to assess the possible influence of covalent DNA modification

Bacteriophage DNA has been reported to be subject to at least ten forms of DNA modification (51–53). RNA broadly has been reported to be subject to >100 forms of covalent modification (82). Both have the potential to disrupt recovery of viral genomes.

As one step toward assessing the effects these modifications, we compared three genotypes of bacteriophage T4: wild-type T4 (termed T4ghmC), T4hmC, and T4C. For wild-type T4, >50% of C residues are modified to glucosyl-hydroxymethylcytosine, which can block attack by host cell nucleases (53, 83, 84). T4hmC is a mutant strain that produces only hydroxymethylcytosine. T4C harbors mutations in genes required for substituting HMC for dCTP and thus only contains unmodified cytosines in genomes. The effects of these modifications were previously documented in DNA sequencing studies as effects on inter-pulse distances in Pacific Biosciences DNA sequencing (53).

To test the effects of these modifications in our Illumina sequencing protocol, we first verified the presence of each DNA modification in the three T4 strains using LC-MS/MS (Fig. S10). We also purified each DNA and showed that sensitivity to restriction enzyme digestion was as expected—that is, DNA containing glucosyl-hydroxymethylcytosine and hydroxymethylcytosine were protected from digestion by Alu I, while DNA containing cytosine was sensitive (Fig. S11). These studies documented the predominance of the expected forms of cytosine in each T4 strain.

**Fig 6:**
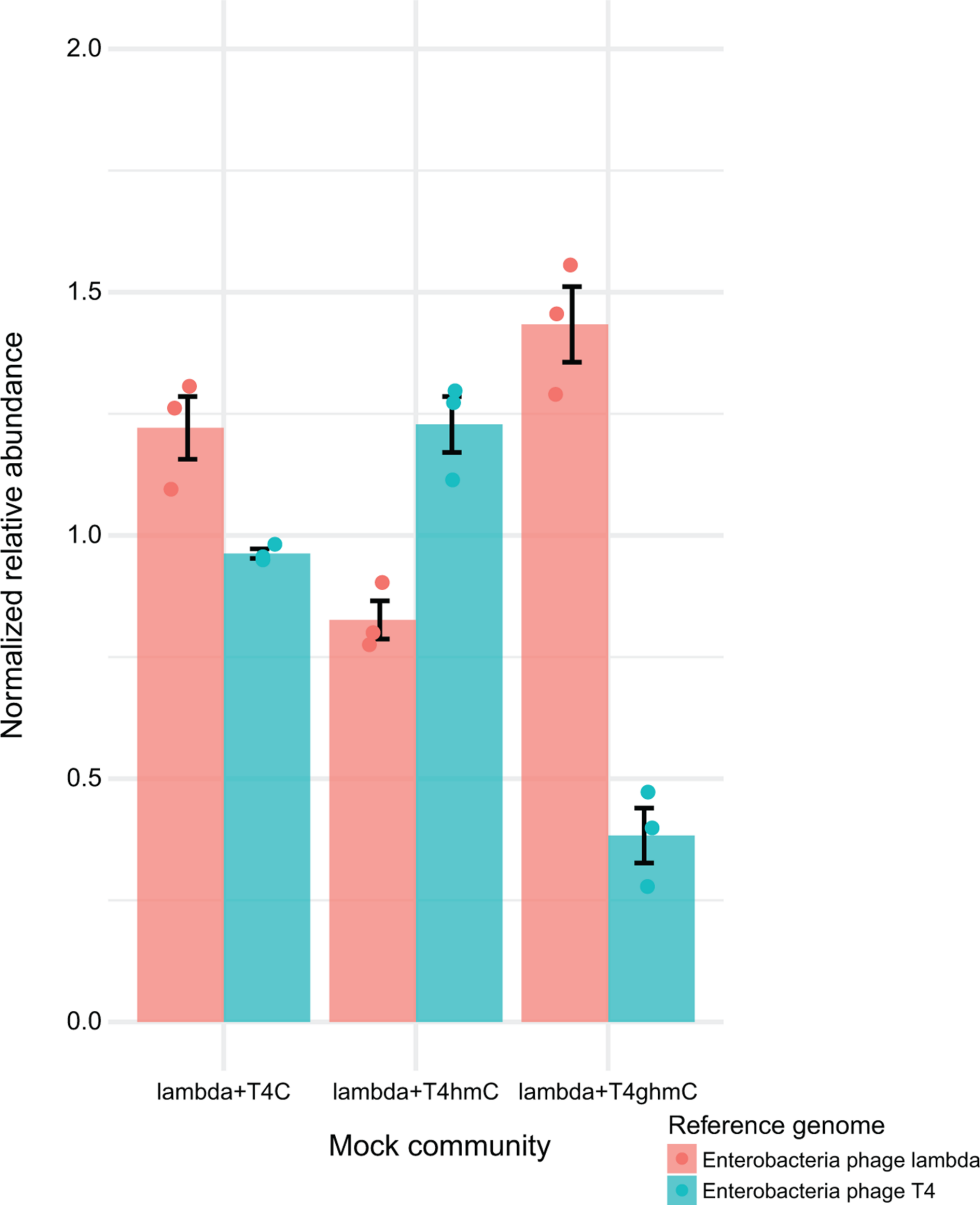
Assessing the potential inhibitory effect of ghmC and hmC on recovery of modified T4 viral genomes. T4 strain with (hmC, ghmC) or without (C) genome base modifications was mixed with lambda, directly extracted for nucleic acid, and subjected to Illumina sequencing. The graph shows the relative abundance of T4 and the lambda control by RPKM from sequencing normalized to that of the input genome copy number from qPCR. Statistical analyses were performed separately for each species. Differences in normalized relative abundance of T4 across groups were assessed using a Kruskal–Wallis test (χ² = 7.2, df = 2, *P* = 0.027). ghmC: glucosyl-hydroxymethylcytosine. hmC: hydroxymethylcytosine.

We then asked whether these modifications disrupted genome sequencing. We mixed each T4 strain with bacteriophage lambda, which lacks the genome modifications mentioned above, and compared recovery after extraction, library preparation and DNA sequencing (Fig. 6). When mixed with lambda, the relative abundance of T4ghmC assayed by sequencing was on average 2.6-fold lower than that expected from the qPCR values for total genomes (Fig. 6; Table S8). For T4C and T4hmC, there was no major reduction in relative abundance by sequencing compared with quantification by qPCR (Fig. 6; Table S8). Thus, hydroxymethylation does not detectably inhibit detection by sequencing, but glucosyl-hydroxymethylation did appear to diminish detection modestly (Kruskal-Wallis, *P* = 0.027). Similar conclusions were reached by Zhai et al. (39). Given that the Nextera method was used to prepare the sequencing library, we conjecture that the transposon-based step used to attach amplification primers may be partially inhibited by glucosyl-hydroxymethylation, because after subsequent amplification steps, the DNA will be unmodified. Thus, the modified genomes were detectable in our samples, though with different efficiencies. It will be useful to document effects of further forms of DNA and RNA modification on virome assays.

## DISCUSSION

Here, we describe steps toward optimizing virome purification methods, in part by taking advantage of tests with a mock community comprised of eight diverse viruses (VirMock1). Previously, multiple groups have published studies of optimizing methods for virome analysis (8, 18, 29–34, 45–47). All of these are valuable, but in any biochemical manipulation, there are typically multiple alternatives at each step, so that the parameter space is large. Here, we start with well-established protocols and work to fill in more of this space with control experiments. In addition, goals in virome studies have been changing over time. Early workers were generally content with recovering large numbers of viruses reproducibly and were less concerned with capturing every possible virus in a community. As the field has matured, interest has grown in the “unknown unknowns,” and method optimization has broadened to include difficult-to-recover viral types that might once have been passed over.

Some of our main conclusions include the following. Multiple published protocols can enrich virus particles from stool and saliva; the usefulness of enrichment steps for low-biomass samples, such as BAL and OP wash, is uncertain. Conventional metagenomic DNA sequencing recovered dsDNA viral genomes, and RNA-seq recovered RNA viral genomes, as expected, but the viral-enrichment protocols greatly increased the fraction of reads attributable to contigs annotated as viral. Amplification methods introduce distortions into the data and should be used with caution. Most viral particles can withstand nuclease treatment, but MS2 was sensitive in some settings; one conjecture is that the structure of the MS2 particle, which includes a portal protein embedded in the icosahedral protein shell, somehow exposes the viral genome to degradation, though other explanations are possible. Longer read Illumina sequencing allowed recovery of longer viral contigs. DNA modifications found in phage T4 genomes did not disrupt capture in the pipelines used, though the presence of glucosyl-hydroxymethylcytosine did reduce recovery modestly.

Based on our assays, we prefer VP1 for analysis of stool samples, and VP4 for analysis of saliva samples based on their overall effectiveness in virus recovery, availability for implementation, and higher throughput. Protocols for each are presented in Document S1. Ongoing work aims to address limitations in current methods, such as potential sample loss during dilutions and handling, and challenges with low-biomass samples.

Short-read and long-read sequencing each have advantages and limitations. Short-reads yield a large amount of data at relatively low cost, but the shorter sequencing length (around 300 bp) presents challenges to microbial genome assembly, especially for sequences with repetitive regions or high-GC content (85). This limitation could be overcome using single-molecule long-read sequencing, as in Oxford Nanopore or PacBio technologies. However, these can be lower-throughput, less accurate, and more expensive (86). In addition, many viral genomes are below 5 kb in length, and so might be removed in steps to purify longer molecules prior to sequence acquisition. As an initial approach to finding a sequencing platform that balances these factors, we compared the Illumina 1,000-cycle kit and 300-cycle kit. The 1,000-cycle kit generated more viral contigs of longer lengths and is suited to capturing short viral genomes. It will be useful to assess the utility of the different methods more fully.

Choices of virome enrichment methods are dependent on the sample type and goals of the experiment. For stool and saliva samples, VP1 and VP2 are reasonable as standard methods; VP3 may be useful in more specialized settings. In studies where small circular single-stranded DNA viruses are the target of interest, it may actually be advantageous to use GenomiPhi to bias recovery in their favor, though GenomiPhi is not optimal under other circumstances. Viral particle enrichment is likely useful for analysis of stool and saliva, where rich viral communities are present along with a complex background. For dilute low-biomass samples, such as BAL, OP wash, and nasopharyngeal swab eluate, losses during handling become major factors. Recovery was better for VirMock1 in stool or saliva, indicating a potential carrier effect of the complex sample background. For low-biomass samples, direct nucleic acid sequencing without enrichment may be best; optimization tests are ongoing. We note that a limitation of this paper is that there are multiple different steps in each protocol, each of which could be carried out in multiple ways, so that not all methods were fully queried; it will be valuable in future studies to explore more of the experimental parameter space.

Multiple additional methods can be used for virome analysis, each of which has strengths and weaknesses. Hybridization capture is one useful method (87), in which viral sequences are enriched from a complex mixture by binding to a library of tagged oligonucleotides. This can be useful when scanning for known viruses, for example, in identifying a virus of human cells responsible for a disease outbreak. This method is not useful for studying viruses that are not complementary to the capture primers, commonly including bacteriophages.

Some of the most extreme variations in viral genomes and related genetic elements were not examined here. Some of the giant viruses (Phylum Nucleocytoviricota) have huge particle sizes and genomes (41), and may be lost during handling. We included vaccinia as a representative of this phylum, but other Nucleocytoviricota are much larger and would be useful to study. At the other extreme, small circular RNAs of only a few hundred bases have been found in diverse sample types (43), some of which may be viruses. Further work to quantify recovery of these elements would be useful.

Sample composition, including the moisture and softness of stool, viscosity of saliva, etc., and effects of storage conditions will also likely influence virome data. Preliminary examination of different storage conditions reveals that processing the stool using VP1 immediately after stool solubilization, after overnight storage at 4°C, or undergoing one round of freeze-thaws from −80°C did not affect the virome enrichment result (data not shown). Further investigation of more parameters would be helpful.

In summary, where possible, it is useful to enrich virome specimens for particles before sequencing to optimize capture of previously unknown viral lineages. This work presents checks on a variety of steps, taking advantage of samples spiked with a community of known composition. However, there are multiple reasonable alternatives at each step; here we review outcomes over a variety of protocol choices, but many more alternative methods could reasonably be considered. It would be useful if multiple groups carrying out such methods optimization studies would publish their work to accelerate further technology refinement.

## MATERIALS AND METHODS

### Human samples

OP wash involved subjects swishing and gargling 10 mL of 1% saline. Saliva was collected (approximately 5 mL) by subjects spitting into a sterile container. Lower respiratory tract samples, specifically BAL, were obtained via bronchoscopy (Protocol #810851). To minimize contamination, upper respiratory tract samples were collected prior to anesthesia, and for bronchoscopy, the first lavage return was discarded.

### Materials and reagents used

Key reagents are listed in Table S9.

### Virome enrichment, sequencing, and analysis methods

Viral particle enrichment was carried out essentially as described. Detailed protocols are in the supplemental material (Methods S1; Tables S10 to S14).

## Data Availability

Sample information and raw sequences are available in the National Center for Biotechnology Information Sequence Read Archive under BioProject ID PRJNA1348896 (Table S15). Intermediate data files are deposited at Zenodo: https://zenodo.org/records/17436149. Raw LC–MS/MS data for DNA nucleoside composition analysis (enzymatic hydrolysis of genomic DNA into individual nucleosides) are deposited in the MassIVE repository under accession number MSV000099555. All bioinformatics scripts used are deposited at GitHub: https://github.com/jduan7/VLP-enrichment-methods-optimization-2025.

## SUPPLEMENTAL MATERIAL

Document S1 msystems.00188-26-s0001.docx Additional protocol details.

Methods S1 msystems.00188-26-s0002.docx Additional experimental details.

Supplemental Figure Legends msystems.00188-26-s0003.docx Legends for Fig. S1 to S11.

Supplemental Figures msystems.00188-26-s0004.pdf Fig. S1 to S11.

Supplemental Tables msystems.00188-26-s0005.xlsx Tables S1 to S15.

ASM does not own the copyrights to Supplemental Material that may be linked to, or accessed through, an article. The authors have granted ASM a non-exclusive, world-wide license to publish the Supplemental Material files. Please contact the corresponding author directly for reuse.
