# Supplementary material for: Optimizing methods for virome analysis based on studies of a synthetic viral community
Source: mSystems. 2026 Jun 2;11(6):e00188-26. doi: 10.1128/msystems.00188-26 (PMC13289072; doi:10.1128/msystems.00188-26)
Supplement: Document S1 — Additional protocol details. [file msystems.00188-26-s0001.docx]

**Supplementary Document S1. Complete workflow of our favored virus enrichment protocols (VP1 and VP4) and recommended methods for processing nucleic acids prior to sequencing**

**VP1: used for enrichment of virus-like particles from stool samples**

Isolation of virus-like particles

1. Thaw 200mg of stool and resuspend in 10mL of SM buffer. Vortex to mix well.
2. Centrifuge the sample at 2,500g for 10min at 4^o^C.
3. Take the supernatant and pass through the 0.22um Sterilflip vacuum PES filter into a new 15mL conical tube.
4. Briefly spin down at 4,000g for 30s.
5. Pre-wash Amicon Ultra-15 Centrifugal Filter by adding 15mL of 0.45um-filtered SM buffer.
   1. SM buffer: 50mM Tris-HCl pH7.5, 100mM NaCl, 8mM MgSO4
6. Spin for 5 mins at 4,000g. Decant the flow through.
7. Transfer the filtrate from step 4 into the Amicon.
8. Spin at 4,000g in 10mins intervals until there are ~250uL remaining in Amicon.
9. Resuspend samples in 10 ml SM buffer and concentrate for the second round (4,000g at 10mins intervals) to a final volume of around 250uL.
   1. The volume that samples could be reconcentrated down to may be variable each time and not necessarily uniform for each sample (usually varying in the range between 100uL to 320uL from our observation). To ensure consistent nuclease treatment, add appropriate volume of SM buffer to top up everything to the same volume as the sample that has the highest volume remaining after two rounds of reconcentration. For the subsequent step, we present an example of topping all sample volumes up to 400uL.
10. Make master mix for DNase & RNase digestion as following:

| Reagent | 1 rxn (uL) |
| --- | --- |
| 10X DNase buffer | 50 |
| Water | 38 |
| RNase | 4 |
| DNase I | 8 |
| Master mix total | 100 |
| Sample | 400 |
| Total | 500 |

1. Incubate at 37^o^C for 30 mins.
2. Add 1uL of 0.5M EDTA into each sample.
3. Incubate the samples at 75C for 10 mins.
4. Proceed to nucleic extraction using QIAamp Viral RNA mini kit on the same day. Store at 4^o^C until use.

**VP4: used for enrichment of virus-like particles from saliva samples**

Pre-filtration of saliva

1. Add 1M DTT to saliva at a final concentration of 25mM.
2. Swirl the tube. Incubate at room temperature for 2 mins.
3. Optional: Centrifuge the sample at 2,500g for 1 mins at 4^o^C. Transfer the supernatant through the 40um strainer that is placed on a new 15mL conical tube. Incubate for 5 mins for saliva to pass through.
4. Store on ice until use.

Isolation of virus-like particles

1. Centrifuge culture supernatant at 2,500g for 10min at 4^o^C.
2. Filter the supernatant through 0.8um PES filter.
   1. Add <600uL supernatant into the Vivaclear.
   2. Centrifuge at 2000g for 6 mins.
3. Pre-wash Amicon Ultra-4 Centrifugal Filter by adding 5mL of 0.45um-filtered SM buffer.
   1. SM buffer: 50mM Tris-HCl pH7.5, 100mM NaCl, 8mM MgSO4
4. Spin for 5 mins at 4,000g. Decant the flow through.
5. Transfer the filtrate from step 2 into the Amicon.
6. Spin at 4,000g in 10mins intervals until there are ~100uL remaining in Amicon.
7. Transfer the sample in the Amicon into an Eppendorf tube.
8. Top up the volume with SM buffer to ~100uL.
9. Make master mix for DNase & RNase digestion as following:

| Reagent | 1 rxn (uL) |
| --- | --- |
| 10X DNase buffer | 14 |
| Water | 23 |
| RNase | 1 |
| DNase I | 2 |
| Master mix total | 40 |
| Sample | 100 |
| Total | 140 |

1. Incubate at 37^o^C for 30 mins.
2. Add 1uL of 0.5M EDTA into each sample.
3. Incubate the samples at 75^o^C for 10 mins.
4. Proceed to nucleic extraction using QIAamp Viral RNA mini kit on the same day. Store at 4^o^C until use.

**Nucleic acid extraction using QIAamp Viral RNA mini kit**

1. Mix 140uL of VLP sample with 560uL of AVL buffer. Vortex.
2. Incubate at rt for 10 min.
3. Briefly spin down.
4. Add 560uL of absolute ethanol to the sample mixture.
5. Mix well by vortexing. Briefly spin down.
6. Add 630uL of the sample mixture into the spin column.
7. Centrifuge at 6,000g for 1 min. Change the collection tube.
8. Repeat step 8-9 until all samples have been loaded.
9. Add 500uL of AW1 buffer to the spin column.
10. Centrifuge at 6,000g for 1 min. Change the collection tube.
11. Add 500uL of AW2 buffer to the spin column.
12. Centrifuge at 20,000g for 3 min. Change the collection tube.
13. Centrifuge at 20,000g for 1 min. Place the spin column in an RNase-free 1.5mL tube.
14. Add 60uL of AVE (elution buffer) to the spin column. Incubate for 1 min.
15. Centrifuge at 6,000g for 1 min.
16. Store viral RNA and DNA at -80^o^C.

**Reverse transcription using the SuperScript IV Kit**

1. Clean the PCR hood and all equipment with RNase-OFF. Repeat cleaning with 70% EtOH.
2. Use RNase-free microcentrifuge tube to prepare the RT-PCR.
3. Make the master mix 1 in a 1.5mL tube.

|  | uL per rxn |
| --- | --- |
| 50uM random hexamers | 1 |
| 10mM dNTPs mix | 1 |
| Water | 1 |
| Total | 3 |

1. Aliquot 3uL of master mix 1 to PCR strip tubes. Add 10uL of the extracted nucleic acid (DNA and RNA).
2. Heat mixture to 65^o^C for 5 mins.
3. After the incubation, briefly centrifuge the tubes and place on ice for > 1 min.
4. Make the master mix 2 in a 1.5mL tube.

|  | uL per rxn |
| --- | --- |
| 5X SSIV buffer | 4 |
| 0.1M DTT | 1 |
| RNaseOUTTM Recombinant RNase Inhibitor | 1 |
| SuperScripteTM IV RT (200U/ul) | 1 |
| Total | 7 |

1. Add 7 uL of master mix 2 to each sample in the PCR strip tubes.
   1. Mix by pipetting gently up and down 10 times and spin down.
2. Incubate the samples using the following condition:
   - - 23^o^C for 10 mins
     - 50 ^o^C for 10 mins
     - 80^o^C for 10 mins
     - Hold at 4^o^C

Keep all samples ON ICE.

**Second-strand cDNA synthesis**

1. Mix well the 20uL of post-RT products with the following components. Centrifuge and spin down.

| Reagent | Volume per rxn (uL) |
| --- | --- |
| 10mM dNTP | 0.5 |
| 50uM random hexamers | 1 |
| H2O | 4.5 |
| Total | 6 |

1. Incubate at 95^o^C for 2mins, then 4^o^C for at least 2mins.
2. Mix well the following components and add to the sample.

| Reagent | Volume per rxn (uL) |
| --- | --- |
| 10X NEBuffer2 | 3 |
| Klenow Fragment DNA polymerase I (5U/uL) | 1 |
| Total | 4 |

1. Incubate at 37^o^C for 1h.
2. Stop the reaction by incubating at 75^o^C for 10mins.
