# Supplementary material for: Optimizing methods for virome analysis based on studies of a synthetic viral community
Source: mSystems. 2026 Jun 2;11(6):e00188-26. doi: 10.1128/msystems.00188-26 (PMC13289072; doi:10.1128/msystems.00188-26)
Supplement: Methods S1 — Additional experimental details. [file msystems.00188-26-s0002.docx]

**Supplementary Methods S1. Detailed methodology for virus propagation, VLP enrichment, sequencing, and bioinformatics analysis.**

Virus propagation

Phage T4, lambda, MS2, and phi6 were propagated in their respective host (Table 1, Table S1), except for T4 C, which was propagated in *E. coli* CR63 first but then had a single plaque picked to infect *E. coli* DH10B [1]. All phage lysates were each made from a single plaque, harvested from liquid culture and treated with chloroform. Plaque assays were performed to determine phage titers. M13 was purchased from AntibodyDesignLaboratories (PH010S) and propagated in *E. coli* C3000 to validate titers. AAV-GFP vectors were prepared as described in Kasimsetty et al.[2]. Briefly, 293T cells were transfected with AAV *trans* plasmid (pAAV-GFP), AAV *cis* plasmid (pAAV2/8), and helper plasmid (pAdDeltaF6), then incubated overnight before washed and incubated for another 48 hours prior to isolation by centrifugation, nuclease treatment, and chloroform treatment. AAV titers were determined by GFP expression measured Incucyte live imaging analysis and by qPCR. Vaccinia virus (strain Western Reserve, ATCC VR-119) was propagated by infecting BSC-1 cells with VV with MOI of 1:10 and then incubating at 37^o^C for 48 hours. Viruses were then harvested by freezing the cell suspension at -80^o^C, repeating freeze-thaws for 3 times followed by sonication of the cells, and taking the supernatant. Murine hepatitis virus was provided by Dr. Susan Weiss. Briefly, MHV was propagated in cultured 17Cl-1 cells and quantified for titers by crystal violet staining.

Plaque assays

Bacterial hosts were cultured overnight. OD600 was measured and then bacterial cultures were mixed with 0.5% top agar and subsequently poured onto LB agar plates. Serial dilutions of phages were then plated onto bacteria in separate spots. Plates were incubated at 37^o^C overnight before counting plaque-forming units. VV was plaqued on BSC-1 cells. Crystal violet staining was used to determine the plaque-forming units (pfu) of VV and MHV.

qPCR

All qPCR assays for reference viruses in VirMock1 were carried out using the TaqMan Fast Virus 1-Step Multiplex Master Mix (No ROX) with the following conditions: 5min at 50^o^C, 20s at 95^o^C and 40 cycles of 3s at 95^o^C and 30s at 60^o^C. qPCRs were performed on a QuantStudio 5 Real-Time PCR System. qPCR probes and primers were ordered from IDT as pre-mixed assays (Table S10).

Plasmids based on pUC57 with PCR amplicons inserted were purchased from GenScript and used as qPCR standards for all viruses except AAV (Table S11). The standard for AAV was trans pAAV-GFP plasmid (Addgene plasmid #32395).

Liquid chromatography-mass spectrometry (LC-MS) analysis to characterize modified T4 DNA.

DNA was extracted from 140 μL of stock T4ghmC, T4hmC and T4C using QIAamp RNA viral mini kit generally following manufacturer’s instructions, except for the use of RNase-free water instead of Buffer AVE for elution. Extracted DNA samples were denatured at 95 °C for 5 min. The resulting single-stranded DNA was digested with nuclease P1 (New England Biolabs, M0660S) in the appropriate digestion buffer at 37 °C for 2 h, followed by digestion with calf intestinal alkaline phosphatase (CIP, New England Biolabs, M0525S) at 37 °C overnight. The digested nucleoside solutions were diluted to a final concentration of 2.5 ng/L. Formic acid was added to all samples to a final concentration of 0.1% prior to LC–MS analysis.

Liquid chromatography was performed on an Ultimate 3000 UPLC system (Thermo Fisher Scientific) using Buffer A (1% acetonitrile, 0.1% formic acid in H₂O) and Buffer B (0.1% formic acid in acetonitrile) at a flow rate of 300 nL/min. One microliter of each sample was loaded onto a reverse-phase C_18_ column (Acclaim PepMap 100, Thermo Fisher Scientific, 164534) maintained at 30°C. The separation was achieved using a segmented linear gradient of Buffer B as follows: 1% from 0–2 min, ramping to 40% at 4 min, increasing rapidly to 95% at 5 min, held at 95% from 5–6 min, and returned to 1% at 7 min for re-equilibration.

Eluted nucleosides were introduced into the mass spectrometer (Exploris 240, Thermo Fisher Scientific) via nano-electrospray ionization using NanoESI emitters (FOSSILIONTECH, The Sharp Singularity) operated at 2500 V. Parallel reaction monitoring (PRM) was applied targeting the nucleosides of interest. Full MS^1^ scans were acquired over an m/z range of 200–500 at 120,000 resolution with a maximum injection time of 100 ms. Targeted precursor ions (10 ppm tolerance) were isolated within a 1 m/z window, then fragmented by higher-energy collisional dissociation (HCD) at 25% normalized collision energy. MS^2^ scans were recorded at 60,000 resolution with a dynamic exclusion time of 8 s.

Raw data were processed using FreeStyle 1.6 (Thermo Fisher Scientific). Target mononucleosides were confirmed by characteristic product ions in the MS^2^ spectra (Table S12). Quantification was performed based on extracted ion chromatogram (XIC) peak areas with a 5 ppm mass accuracy threshold (Table S13). To compare the relative abundance of modified/unmodified cytosine across the samples, normalization was performed using the signal of 2′-deoxythymidine (dT) to correct for differences in sample loading (Table S13 and Fig. S9). For each nucleoside, normalized relative abundance was calculated as:

$$Relative \%= \frac{{Signal}_{sample}}{max({Signal}_{all samples})} \times100$$

Characterization of modified T4 DNA with by digestion with the restriction endonuclease Alu I

DNA samples of T4ghmC, T4hmC, and T4C were extracted the same way as that mentioned above for LC-MS. Extracted DNA was digested with C-specific AluI restriction enzymes at 37°C for 1 hour and then visualized on 1% agarose gel electrophoresis with ethidium bromide.

Assembling the mock viral community

Over the course of the experiments, multiple batches of VirMock1 were assembled. VirMock1 was prepared by mixing T4 ghmC, lambda, MS2, phi6, M13, MHV, AAV vector, and VV. Mixtures were then stored in single-use aliquots at -80^o^C. See Table S4 for VirMock1 composition of each batch as quantified by qPCR. Concentrations in VirMock1 were designed in reference to Neto et al. so that 1) the virus copy numbers would reach a level putatively detectable by sequencing and 2) the VirMock1 aliquot could be maintained at a volume lower than ~1/3 of the total volume to ensure that the sample content would not be changed dramatically by being spiked with VirMock1.

Nuclease treatment and nuclease titration experiment on VirMock1

All samples treated with nuclease in this paper underwent 1X nuclease treatment unless otherwise specified. 1X nuclease included 10X DNase buffer (Roche, 4716728001), 2 μL of Roche DNase I (Roche, 4716728001) and 1 μL of RNase (Roche, 11119915001) for every 100 μL of post-virome prep sample. Nuclease digestion was carried out at 37^o^C for 30 minutes, followed by adding 1 μL of 0.5M EDTA and incubating at 75^o^C for 10 minutes to inactivate the nucleases.

Nuclease titration experiment using mock community only: Mock community was treated with 0.1X nuclease (10-fold dilution of the DNase I and RNase used in 1X nuclease), 1X nuclease, 10X nuclease (10-fold concentration of the DNase I and RNase used in 1X nuclease), or no nuclease.

Nuclease titration experiment using the mock community spiked into stool: The mock community was spiked into 200mg of solubilized stool, passed through VP1, with the nuclease treatment step being 0.1X, 1X, 10X, or no nuclease.

Nuclease titration on free nucleic acid

Mouse b2m DNA (accession: NM_009735.3) and Rat total RNA (accession: NM_017015.3) were spiked into VirMock1 in a total volume of 150 μL and passed through VP4 with different titrations of nuclease (none, 0.01X, 0.1X, and 1X nuclease). qPCR assays were performed to quantify the amount of mouse b2m DNA and rat total RNA after treatment (Table S5). IDT qPCR assay Rn. PT39a.22214822.g and Mm.PT.39a.22214835 were used for quantifying Rat total RNA and Mouse b2m DNA, respectively. Rat XpressRef Universal Total RNA was used as qPCR standard for rat total RNA. See Table S10 and S11 for the qPCR assay used for mouse b2m DNA.

Base modification experiment

Lambda was mixed with different T4 strains, including T4 ghmC, T4 hmC, and T4 C, in proportions provided in Table S8, and then extracted using the QIAamp Viral RNA kit. The extracted nucleic acid was prepared for sequencing using Nextera XT library prep and sequenced using Nextseq1000 (300-cycles).

Saliva amplification experiment

3.5 mL of pooled saliva was treated with 1M DTT for a final concentration of 25mM DTT, incubated at room temperature for 2 min, then centrifuged at 2500g for 1 min at 4^o^C. The supernatant was passed through a 40um strainer (pluriStrainer Mini 40uM, 43-10040-40). 394uL of saliva aliquot or equal volume of SM buffer was then spiked or not spiked with VirMock1, and topped up to a final volume of 600uL with SM buffer. The samples were centrifuged at 2,500g for 10min at 4^o^C. The supernatant was filtered through a 0.8um pore-size PES filter (Sartorius, VK01P042) by centrifuging at 2000g for 6 mins. The filtrate was transferred into Amicon Ultra-4 Centrifugal Filters (Thermo Fisher Scientific, UFC810096) and spun at 4,000g in 10mins intervals until there was ~100uL remaining in the Amicon. The concentrate was then treated with 1X nuclease as described above. A total of 140μl VLP preparation was used for viral nucleic acid extraction immediately after nuclease treatment using QIAamp Viral RNA kit. For direct extraction samples, VirMock1 was resuspended in 394uL of SM buffer, treated with 1X nuclease, and extracted for nucleic acid. Extracted nucleic acid was stored at -80^o^C until use.

Total nucleic acid was either reverse transcribed and amplified using the Complete Whole Transcriptome Amplification kit (MilliporeSigma, WTA2) following a protocol from Conceicao-Neto et al.[3], or reverse transcribed using SuperScriptIII (reverse transcription methods described below) and then amplified with GenomiPhiV3 (Cytiva 25-6601-24) or MALBAC (Yikon Genomics, KT110700110) or ResolveDNA Whole Genome Single-Cell Core Kit (BioSkryb Genomics) following manufacturers’ instructions. 17 cycles were applied for MALBAC and WTA2. The PCR products were purified using QIAquick PCR purification kit and stored at -20^o^C until use. Samples were sequenced using Illumina Nextera XT library prep followed by sequencing on NextSeq2000, except for samples undergoing PTA, which used the library prep from the manufacturer following manufacturer’s instructions and subsequently sequenced on a Illumina MiniSeq.

Stool solubilization

Frozen stool was thawed processed as described by Dillon et al. 2021[4]. Briefly, stool was diluted 1:1 with dPBS in a 50 ml conical tube with twelve 1 mm glass beads (Thermo Fisher Scientific, AC465941000) before being mixed vigorously for at least 30 seconds or until homogenized. Materials were strained using a 500um cell strainer (VWR International, 43-50500-03) before being pooled in a sterile container pooling more than 5 donor fecal samples together.

Methods for purification of virus-like particles in stool spike-in experiment

VP1: this protocol follows the protocol published by Liang et al. [5] . Briefly, 50uL of VirMock1, 200mg of solubilized stool, or 200mg of solubilized stool spiked with 50uL of VirMock1, was resuspended in SM buffer (50 mM Tris-HCl pH 7.5, 100 mM NaCl, 8 mM MgSO_4_) to reach a final volume of 10mL. Samples were spun down and filtered through a 0.22-μm-pore-size vacuum filter (Thermo Fisher Scientific, SCGP00525). The filtrate was concentrated using a 100-kDa-molecular-mass Amicon Ultra-15 Centrifugal Filter (Thermo Fisher Scientific, UFC910096), resuspended in 10 ml SM buffer and concentrated for the second time to a final volume of around 250μl. The concentrate was topped up to 400 μL using SM buffer and then treated with 1X nuclease as described above. A total of 500μl VLP preparation was used for viral nucleic acid extraction immediately after nuclease treatment using QIAamp Viral RNA kit (per manufacturer, extracts total nucleic acids). Extracted nucleic acid was stored at -80^o^C until use.

VP2: This protocol was adapted from Conceicao-Neto et al. [3]. Briefly, 50uL of VirMock1, 50mg of solubilized stool, or 50mg of solubilized stool spiked with 50uL of VirMock1, was resuspended in SM buffer to reach a final volume of 500 μL. Samples were then centrifuged at 17,000g for 3 mins. The supernatant was passed through a 40um pore-size strainer (pluriStrainer Mini 40uM, 43-10040-40) and then a 0.8um pore-size PES filter (Sartorius, VK01P042). The filtrate was topped up to 500 μL using SM buffer and then treated with 1X nuclease as described above. A total of 600μl VLP preparation was used for viral nucleic acid extraction immediately after nuclease treatment using QIAamp Viral RNA kit (per manufacturer, extracts total nucleic acids). Extracted nucleic acid was stored at -80^o^C until use.

VP3: this protocol follows Shkoporov et al. [6]. Briefly, 50uL of VirMock1, 500 mg of solubilized stool, or 500 mg of solubilized stool spiked with 50uL of VirMock1, was resuspended in SM buffer to reach a final volume of 10mL. The resuspended sample was homogenized by vigorous vortexing, centrifuged twice, then filtered twice through a 0.45 um pore PES syringe-mounted membrane filters (Millipore, SLHPR33RS). NaCl and PEG-8000 powders were added to reach a final concentration of 0.5M and 10% w/v respectively. Samples were incubated overnight. On the next day, samples were centrifuged to harvest pellets, which were then resuspended in 400 μL SM buffer and equal volume of chloroform to extract. Emulsions were then centrifuged at 2500g for 5 min to harvest the aqueous phase, which was then mixed with 40 μl of a solution of 10 mM CaCl_2_ and 50 mM MgCl_2_, treated with 8U TURBO DNase (Ambion/ThermoFisher Scientific, AM2238) and 20 U of RNase I (ThermoFisher Scientific, EN0601), and incubated at 37^o^C for 1h followed by 10 min at 70^o^C. Proteinase K (40 μg) and 20 μl of 10% SDS was then added to the tubes and incubated for 20 min at 56 °C, followed by addition of 100 μl of Phage Lysis Buffer (4.5 M guanidinium isothiocyanate, 44 mM sodium citrate pH 7.0, 0.88% sarkosyl, 0.72% 2-mercaptoethanol) and incubation at 65^o^C for 10 min. The lysates were extracted twice by vortexing with equal volume of Phenol/Chloroform/Isoamyl Alcohol 25:24:1 (Fisher Scientific, 15693031) and centrifugation at 8000 g for 5 min at room temperature. The nucleic acid was purified from the aqueous phase using DNeasy Blood & Tissue Kit (Qiagen, 69504) (per manufacturer, extracts total nucleic acids).

Direct extraction: 50uL of VirMock1 was resuspended in 350uL SM buffer, treated with 1X nuclease as described above, and extracted for total nucleic acid using QIAamp Viral RNA mini kit (per manufacturer, extracts total nucleic acids).

VLP preparation from saliva, OP wash, and BAL (VP4) in liquid sample spike-in experiment

Pooled saliva was pre-treated with 1M DTT for a final concentration of 25mM DTT and incubated at room temperature for 2 min. Pooled OP wash and BAL was directly thawed on ice. Then, 43uL of VirMock1, 557uL of saliva or OP wash or BAL, or 557uL of saliva or OP wash or BAL spiked with 43uL of VirMock1, was resuspended in SM buffer to reach a final volume of 600 μL. Samples were then centrifuged at 2,500g for 10min at 4^o^C. The supernatant was filtered through a 0.8um pore-size PES filter (Sartorius, VK01P042) by centrifuging at 2000g for 6 mins. The filtrate was transferred into Amicon Ultra-4 Centrifugal Filter (Thermo Fisher Scientific, UFC810096) and spun at 4,000g in 10mins intervals until there were ~100 μL remaining in the Amicon. The concentrate was then treated with 1X nuclease as described above. For direct extraction samples, 43uL of VirMock1 was resuspended in SM buffer to reach a final volume of 100uL and treated with 1X nuclease. A total of 140μl VLP preparation of each sample was then used for viral nucleic acid extraction immediately after nuclease treatment using the QIAamp Viral RNA kit. Extracted nucleic acid was stored at -80^o^C until use. For samples undergoing PTA, 0.3ng DNA was used following the manufacturer’s instructions. PCR product was purified using QIAquick PCR purification kit according to the manufacturer’s instructions and stored at -20^o^C until use.

Extraction and reverse transcription of viral nucleic acids

DNA and RNA was extracted from purified VLPs using QIAamp Viral RNA extraction kit (Qiagen, 52904) following the manufacturer’s instructions. Reverse transcription was then performed on total nucleic acid using SuperScript III (ThermoFisher Scientific) following the protocol in Marques et al[7]. Downstream sequence analysis was performed on DNA and cDNA together.

Reverse transcription and second-strand synthesis of VirMock1 community

VirMock1 community was directly extracted for total nucleic acid. Reverse transcription was then carried out using either SuperScript III or SuperScript IV, with condition RT 1 performed as described above using SuperScript III, condition RT 2 performed using SuperScript III following manufacturer’s instruction, and condition RT 3 performed using SuperScript IV following manufacturer’s instructions. cDNA second-strand synthesis was performed by mixing 20uL of reverse transcription product with 0.5uL of 10mM dNTPs Mix (ThermoFisher), 1uL of 50uM random hexamers (ThermoFisher), and 4.5uL of molecular-grade water, incubated at 95°C for 2 minutes and 4°C for 2 minutes, followed by adding 3uL of 10X NEBuffer2 and 5 units of Klenow DNA Polymerase I (New England Biolabs #M0210L) and incubation at 37°C for 1 hour. The reaction was stopped by incubating the samples at 75°C for 10 minutes. dsDNA products were purified using QIAquick PCR purification kit according to the manufacturer’s instructions for a final elution volume of 30uL.

Illumina 300-cycles sequencing

All sequencing was performed using Illumina sequencing machines. The Nextera XT library preparation kit (FC-131-1096) was used for all samples unless otherwise specified. Libraries were tagged using IDT DNA/RNA UD Indexes and quantified with the Quant-iT PicoGreen dsDNA assay. Libraries were sequenced on the same NextSeq 2000 instrument using P1 300-cycle reagents. All libraries received a 1% PhiX spike-in.

Illumina 1000-cycles sequencing

The library pool as prepared for 300-cycle sequencing had an average fragment size of 542 bp. A 0.55X bead clean-up was performed on the pool using AMPure XP beads (Beckman-Coulter) to target larger fragments and bring the average fragment size closer to 1000 bp. The pool was sequenced on a 2x500 cycle kit on the Illumina MiSeq i100 (the only compatible instrument at the time of the experiment).

Whole DNA metagenomic sequencing

DNA was extracted from approximately 200 μL total of starting material using the Qiagen DNeasy PowerSoil Pro kit. Extracted DNA was quantified using the Quant-iT PicoGreen dsDNA assay kit (Thermo Fisher Scientific). Shotgun libraries were generated from 7.5 ng DNA using the Illumina DNA Prep Library Prep kit and IDT for Illumina unique dual indexes at 1:4 scale reaction volume. The Quant-iT PicoGreen dsDNA assay kit was used to assess library success. An equal volume of library was pooled from every sample and sequenced using a 300-cycle Nano kit on the Illumina MiSeq i100. Libraries were then re-pooled based on the demultiplexing statistics of the MiSeq Nano run. The final library pool was QC’ed on the Qiagen QIAxcel with the QIAxcel DNA High Resolution kit to check fragment size distribution and absence of adaptor fragments. Libraries were sequenced on an Illumina Novaseq X flow cell, producing 2x150 bp paired-end reads. Extraction blanks and nucleic acid-free water were processed along with experimental samples to empirically assess environmental and reagent contamination. A laboratory-generated mock community consisting of DNA from *Vibrio campbellii* and Lambda phage was included as a positive sequencing control.

RNA-seq Analysis

RNA was extracted from approximately 200 μL total of starting material using the Qiagen RNeasy PowerFecal Pro kit. RNA was quantified using the Qubit™ RNA HS Assay Kit (Thermo Fisher Scientific). RNA quality and RIN were checked with the Agilent TapeStation RNA ScreenTape Analysis kit. Libraries were generated from 25-500 ng RNA using the Illumina Stranded Total RNA Prep, Ligation with Ribo-Zero Plus Microbiome and Illumina RNA UD indexes. Library success was assessed by the Quant-iT PicoGreen dsDNA assay kit. Libraries were QC’ed on the Qiagen QIAxcel with the QIAxcel DNA High Resolution kit to check fragment size distribution and absence of adaptor fragments. Libraries were then diluted to the same molar concentration and pooled in equal amounts. Libraries were sequenced on an Illumina Novaseq X flow cell, producing 2x150 bp paired-end reads. Extraction blanks and nucleic acid-free water were processed along with experimental samples to empirically assess environmental and reagent contamination. A positive sequencing control was used consisting of *E. coli* total RNA (Thermo Fisher Scientific).

Bioinformatic analysis

Sequences were analyzed using the bioinformatics pipeline Sunbeam v4.0.0, comprised of adapter trimming (Trimmomatic), quality filtering (Komplexity), taxonomic assignment (Kraken), contig assembly (Megahit v1.2.9), ORF prediction (Prodigal), and virus identification and annotation (Cenote-Taker2 v2.1.3[8], Cenote-Taker3 Database 3.1.1: https://github.com/mtisza1/Cenote-Taker3). For verification of virus annotation results by Cenote-Taker2, geNomad (version 1.7.1)[9] was run on selected samples using the end-to-end command. To calculate the relative abundance of each virus in the mock community, all reads were trimmed to remove adaptors and filtered with phred quality ≥Q30 using fastp, mapped using Burrows-Wheeler Aligner (BWA) against the reference genomes of all VirMock1 community members (Table S14), and analyzed for the number of mapped reads per kilobase of genome target per million sample reads. The percentage of viral reads was calculated by dividing the total number of sample reads by the number of reads that were mapped to the viral contigs identified by the annotation tool used. Comprehensive assignment of any bacteria, viruses, or animal content in the VirMock1 samples was performed by Kraken2 using the “core_nt” pre-built kraken2 database which includes GenBank, RefSeq, TPA, and PDB (released October 15, 2025). All visualizations were done in RStudio (version 2025.05.1+513) and processed by Illustrator.

Statistical Analyses

All statistical tests were carried out in R (version 4.5.1). PCoA analyses were performed using Bray-Curtis distances computed based on relative abundance of VirMock1 reference virus in each sample using the vegan package (version 2.7-1). Within-group distances were grouped by usedist (version 0.4.0) package. Variation in community composition across the tested condition groups were assessed with the tested condition as a categorical predictor using PERMANOVA in the vegan package (version 2.7-1). All other statistical tests were performed using base R.

**References**

1. Bryson AL, Hwang Y, Sherrill-Mix S, Wu GD, Lewis JD, Black L, Clark TA, Bushman FD: **Covalent Modification of Bacteriophage T4 DNA Inhibits CRISPR-Cas9.** *mBio* 2015, **6:**e00648.

2. Kasimsetty A, Hwang Y, Everett JK, McFarland AG, Zolnoski SA, Lu T, Roche AM, Martinez-Garcia PM, Sabatino DE, Bushman FD: **Modulation of AAV transduction and integration targeting by topoisomerase poisons.** *Mol Ther Methods Clin Dev* 2024, **32:**101364.

3. Conceicao-Neto N, Zeller M, Lefrere H, De Bruyn P, Beller L, Deboutte W, Yinda CK, Lavigne R, Maes P, Van Ranst M, et al: **Modular approach to customise sample preparation procedures for viral metagenomics: a reproducible protocol for virome analysis.** *Sci Rep* 2015, **5:**16532.

4. Dillon KCH, J. R.; Aspinwall, B. A.; Lucking, S. T.; Carleton, H.; Huang, A. D.; Williams-Newkirk, A. J.: **Small-Scale Stool Homogenization.** *protocolsio* 2021.

5. Liang G, Zhao C, Zhang H, Mattei L, Sherrill-Mix S, Bittinger K, Kessler LR, Wu GD, Baldassano RN, DeRusso P, et al: **The stepwise assembly of the neonatal virome is modulated by breastfeeding.** *Nature* 2020, **581:**470–474.

6. Shkoporov AN, Ryan FJ, Draper LA, Forde A, Stockdale SR, Daly KM, McDonnell SA, Nolan JA, Sutton TDS, Dalmasso M, et al: **Reproducible protocols for metagenomic analysis of human faecal phageomes.** *Microbiome* 2018, **6:**68.

7. Marques AD, Sherrill-Mix S, Everett JK, Adhikari H, Reddy S, Ellis JC, Zeliff H, Greening SS, Cannuscio CC, Strelau KM, et al: **Multiple Introductions of SARS-CoV-2 Alpha and Delta Variants into White-Tailed Deer in Pennsylvania.** *mBio* 2022, **13:**e0210122.

8. Tisza MJ, Belford AK, Dominguez-Huerta G, Bolduc B, Buck CB: **Cenote-Taker 2 democratizes virus discovery and sequence annotation.** *Virus Evol* 2021, **7:**veaa100.

9. Camargo AP, Roux S, Schulz F, Babinski M, Xu Y, Hu B, Chain PSG, Nayfach S, Kyrpides NC: **Identification of mobile genetic elements with geNomad.** *Nat Biotechnol* 2024, **42:**1303–1312.
