# Supplementary material for: Optimizing methods for virome analysis based on studies of a synthetic viral community
Source: mSystems. 2026 Jun 2;11(6):e00188-26. doi: 10.1128/msystems.00188-26 (PMC13289072; doi:10.1128/msystems.00188-26)
Supplement: Supplemental Figure Legends — Legends for Fig. S1 to S11. [file msystems.00188-26-s0003.docx]

**SUPPLEMENTAL FIGURE LEGENDS**

Figure S1. Relative abundance of class of viruses as annotated by Cenote-Taker2 in A, D & F) saliva, B, E, G-I) stool or C) BAL that was analyzed by direct extraction of A & B) DNA or C-E) RNA, or by viral particle enrichment using F) VP4, G) VP1, H) VP2, or I) VP3. The identification of Megaviricetes in panel E appears to be erroneous: a few short contigs that are likely bacterial genome fragments were classified as Mimiviridae by Cenote-Taker2; since relative abundance was calculated based on RPKM using the length of the identified viral contig, the abundance of Megaviricetes resulted in a large proportion of the viral contents of the sample. We include this as a cautionary note.

Figure S2. Comparison of viral sequence recovery after virus particle enrichment followed by sequencing, versus direct metagenomic sequencing of total DNA or RNA. A-D) Percentages of reads mapped to viruses annotated by geNomad in stool that was analyzed by A) direct extraction of DNA (red) or RNA (blue), or viral particle enrichment using the B) VP1, C) VP2, or D) VP3 protocols (green), with the mean of triplicate measurements represented by a black bar. E-H) Percentages of reads mapped to viruses annotated by geNomad in E & F) BAL or G & H) saliva that was analyzed by E & G) direct extraction and metagenomic DNAseq (red) and RNAseq (blue), or by F & H) viral particle enrichment using the VP4 protocol (green), with the mean of triplicate measurements represented by a black bar.

Figure S3. A-E) Percentage of reads mapping to viruses from VirMock1 or any de novo assembled viral contigs after VirMock1 was spiked into A) BAL, B) OP wash, C) saliva, or D-E) SM buffer. Samples were A-D) enriched for viral particles using VP4 or E) nuclease treated and then directly extracted for nucleic acid. Reads aligning to any virus as annotated by Cenote-Taker2 are indicated in tan; reads annotating as aligning to a virus in VirMock1 are shown in green; the mean of triplicate measurements is represented by the black bar. Dots below the limit of detection represent samples with no viral contigs detected.

Figure S4. Comparison of the effects of direct extraction and different virus enrichment methods on VirMock1 viral genome recovery from SM buffer and stool. PCoA was performed using Bray-Curtis distances computed based on relative abundance of VirMock1 reference virus in each sample. Variation in community composition across different treatments were assessed using PERMANOVA on the Bray-Curtis distances (R^2^=0.456, p=0.001). Ellipses showing the 95% confidence interval around the group mean were drawn for treatment groups with more than 3 samples, assuming a multivariate t-distribution.

Figure S5. Relative abundance of classes of viruses as annotated by Cenote-Taker2 in stool spiked with VirMock1 after enrichment for viral particles using A) VP1, B) VP2, or C) VP3.

Figure S6. Relative abundance of species as classified by Kraken2 in A, B, D, F, H & L) SM buffer, C, E & G) stool, I) BAL, J) OP wash, or K) saliva spiked with VirMock1 community that was analyzed by A & H) nuclease treated and directly extracted nucleic acid, or viral particle enrichment using B & C) VP1, D & E) VP2, F & G) VP3, or I-L) VP4.

Figure S7. Relative abundance of each VirMock1 reference virus after VirMock1 was spiked into A & B) BAL, C & D) OP wash, E & F) saliva, or G & H) SM buffer and followed by VP4, or I & J) spiked into SM buffer, nuclease treated and directly extracted; extracted nucleic acids were then A, C, E, G & I) amplified by PTA or B, D, F, H & J) remained unamplified.

Figure S8. Comparison of effect of different reverse transcription and second-strand cDNA synthesis protocols on VirMock1 viral genome recovery. Samples (without nuclease treatment) were directly extracted for total nucleic acid, reverse transcribed under condition RT1, RT2, or RT3, and proceeded to Illumina sequencing with or without second-strand cDNA synthesis and dsDNA purification (Supplementary Methods S1). Each condition was performed in triplicate twice. Relative abundance of VirMock1 community (A-G) and percentage of reads mapping to viruses from VirMock1 (H-N) after different treatments: A & H) using SSIII under RT condition 1, B & I) using SSIII under RT condition 2, C & J) using SSIII under RT condition 2 followed by second-strand cDNA synthesis, D & K) using SSIII under RT condition 2 followed by second-strand cDNA synthesis and dsDNA purification, E & L) using SSIV under RT condition 3, F & M) using SSIV under RT condition 3 followed by second-strand cDNA synthesis, G & N) using SSIV under RT condition 3 followed by second-strand cDNA synthesis and dsDNA purification. SSIII stands for SuperScript III. SSIV stands for SuperScript IV. Klenow stands for second-strand cDNA synthesis via DNA polymerase I Large (Klenow) fragment. Reads aligning to any virus as annotated by Cenote-Taker2 are indicated in tan; reads annotating as aligning to a virus in VirMock1 are shown in green; the mean of replicates measurements is represented by the black bar. Size of the circle indicates the total number of reads of the sample. O) Within-group Bray-Curtis dissimilarities of VirMock1 reference virus relative abundance profiles were compared between Klenow and no-Klenow treatment groups using linear regression (two-sided t test, p<2.22e-16).

Figure S9. Distribution of samples spiked with VirMock1 by average viral contig lengths and sequencing depth (300-cycle kit). Each point corresponds to a sample with detectable viral contigs, with error bars representing the standard errors of the mean viral contig length. Trends were estimated using linear regression of log_10_-transformed mean viral contig length against log_10_-transformed sequencing depth (two-sided t test, p=1.8e-16). Different colors represent the experiments that the samples are from.

Figure S10. Validation of DNA modifications in T4 strains by LC-MS. Relative abundance of each modified and unmodified nucleoside in T4 C, T4 hmC, and T4 ghmC determined by LC-MS are shown in the bar graph.

Figure S11. Validation of T4 strains by AluI digestion of T4 DNA. Phage T4 ghmC, T4 hmC and T4 C DNA left untreated (-) or treated (+) with the restriction enzyme AluI, which cleaves unmodified DNA.
