# Supplementary material for: Optimizing methods for virome analysis based on studies of a synthetic viral community
Source: mSystems. 2026 Jun 2;11(6):e00188-26. doi: 10.1128/msystems.00188-26 (PMC13289072; doi:10.1128/msystems.00188-26)

Figure S1

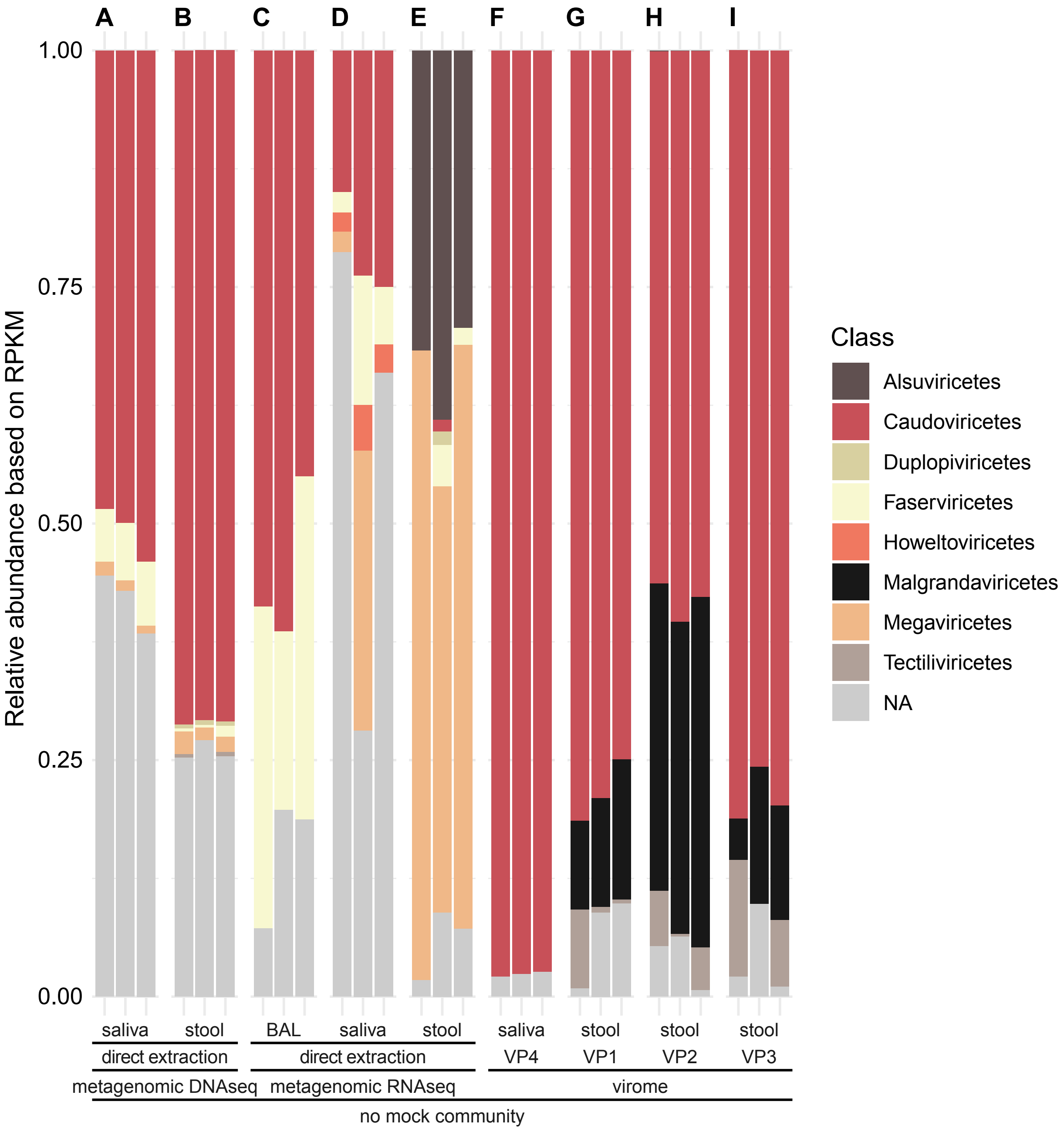
A
B
C
D
E
F
G
H
I
Relative abundance based on RPKM
1.00
0.75
0.50
0.25
0.00
saliva
stool
BAL
saliva
stool
saliva
stool
stool
stool
direct extraction
direct extraction
VP4
VP1
VP2
VP3
metagenomic DNAseq
metagenomic RNAseq
virome
no mock community
Class
Alsuviricetes
Caudoviricetes
Duplopiviricetes
Faserviricetes
Howeltoviricetes
Malgrandaviricetes
Megaviricetes
Tectiliviricetes
NA

Figure S2

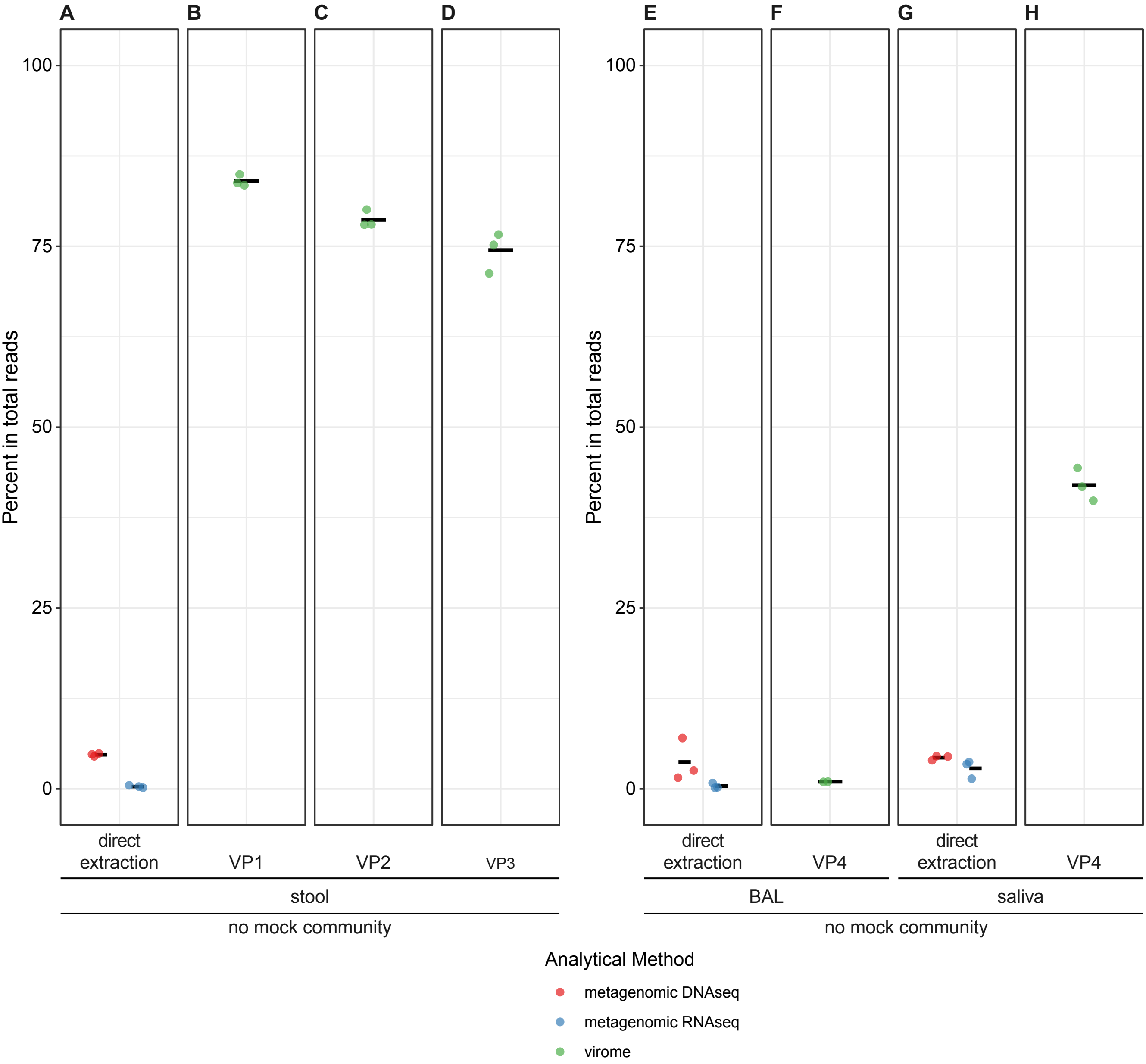

A
B
C
D
E
F
G
H
Percent in total reads
100
75
50
25
0
direct extraction
VP1
VP2
VP3
VP4
direct extraction
VP4
stool
BAL
saliva
no mock community
no mock community
Analytical Method
metagenomic DNAseq
metagenomic RNAseq
virome

Figure S3

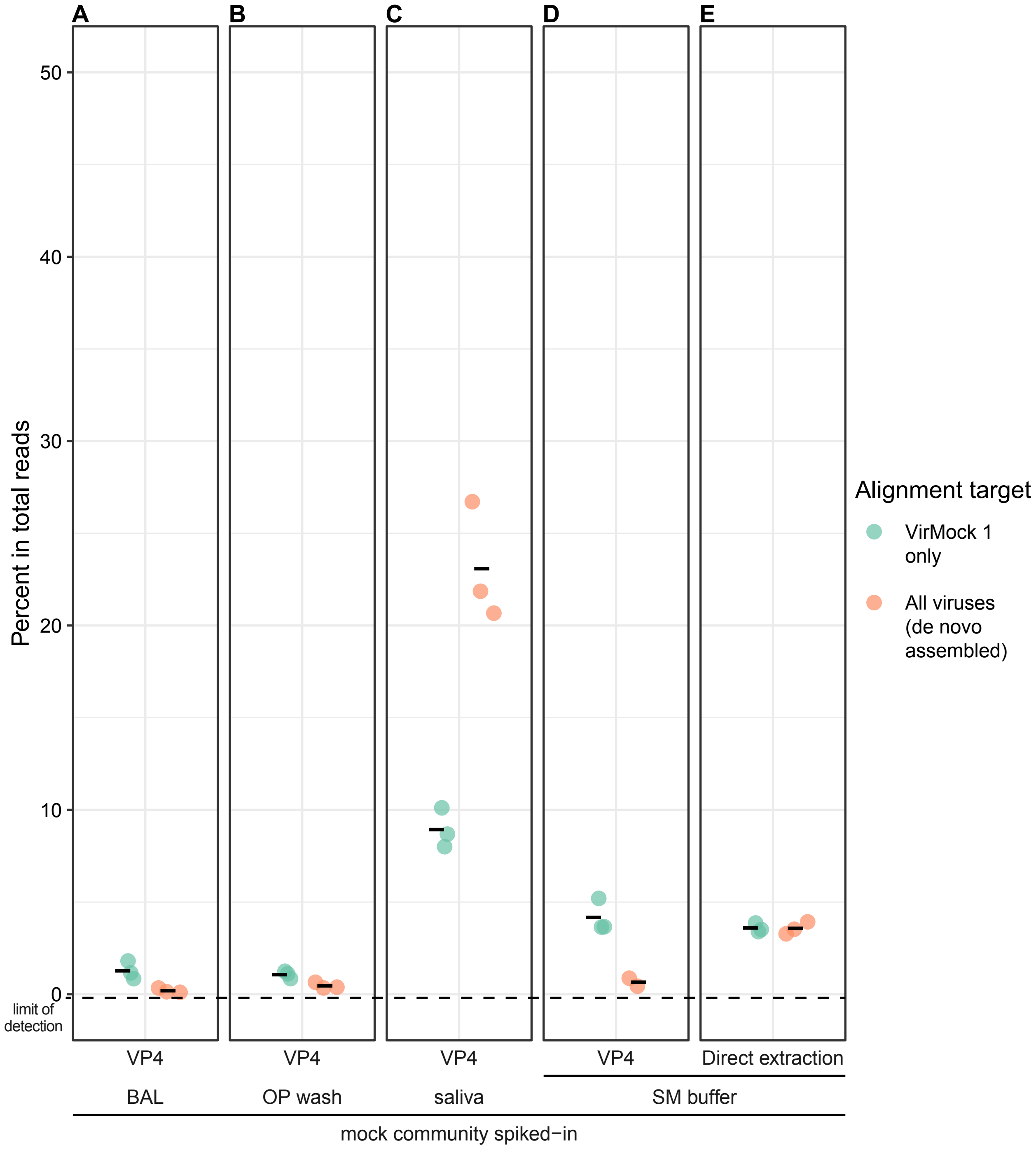
A
B
C
D
E
Percent in total reads
50
40
30
20
10
0
limit of detection
VP4
VP4
VP4
VP4
Direct extraction
BAL
OP wash
saliva
SM buffer
mock community spiked−in
Alignment target
VirMock 1 only
All viruses (de novo assembled)

Figure S4

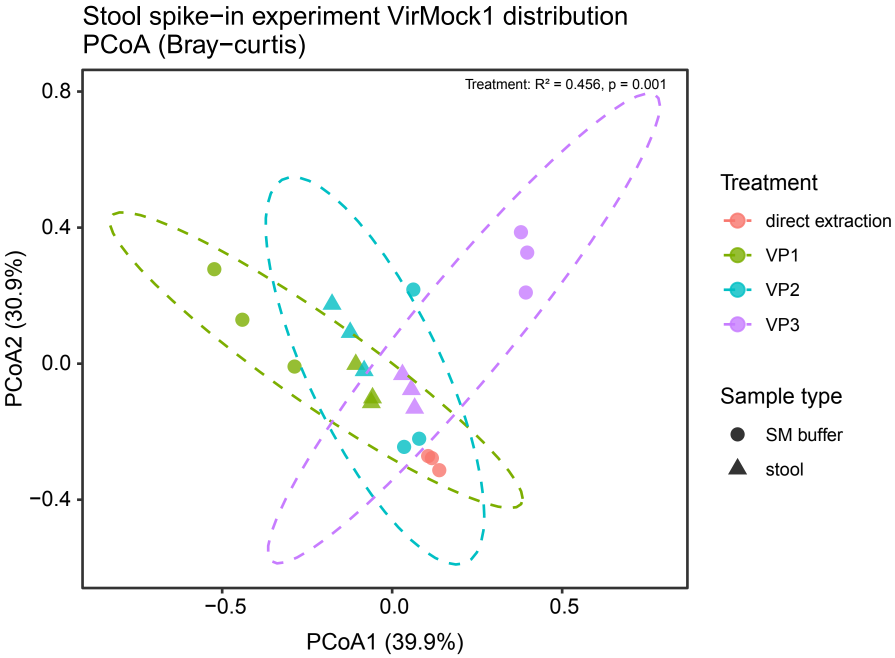
Stool spike−in experiment VirMock1 distribution
PCoA (Bray−curtis)
Treatment: R² = 0.456, p = 0.001
0.8
0.4
0.0
−0.4
PCoA2 (30.9%)
−0.5
0.0
0.5
PCoA1 (39.9%)
Treatment
direct extraction
VP1
VP2
VP3
Sample type
SM buffer
stool

Figure S5

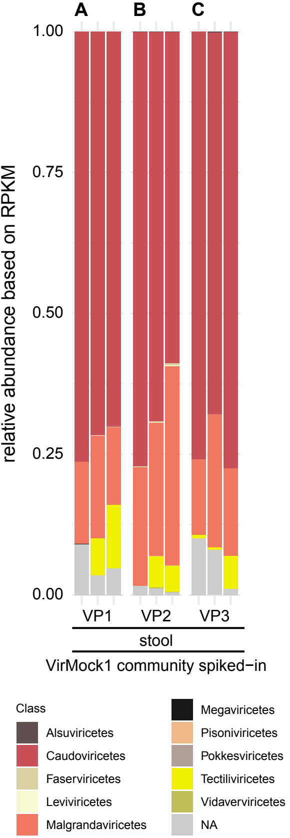

A
B
C
1.00
0.75
0.50
0.25
0.00
relative abundance based on RPKM
VP1
VP2
VP3
stool
VirMock1 community spiked−in
Class
Alsuviricetes
Caudoviricetes
Faserviricetes
Leviviricetes
Malgrandaviricetes
Megaviricetes
Pisoniviricetes
Pokkesviricetes
Tectiliviricetes
Vidaverviricetes
NA

Figure S6

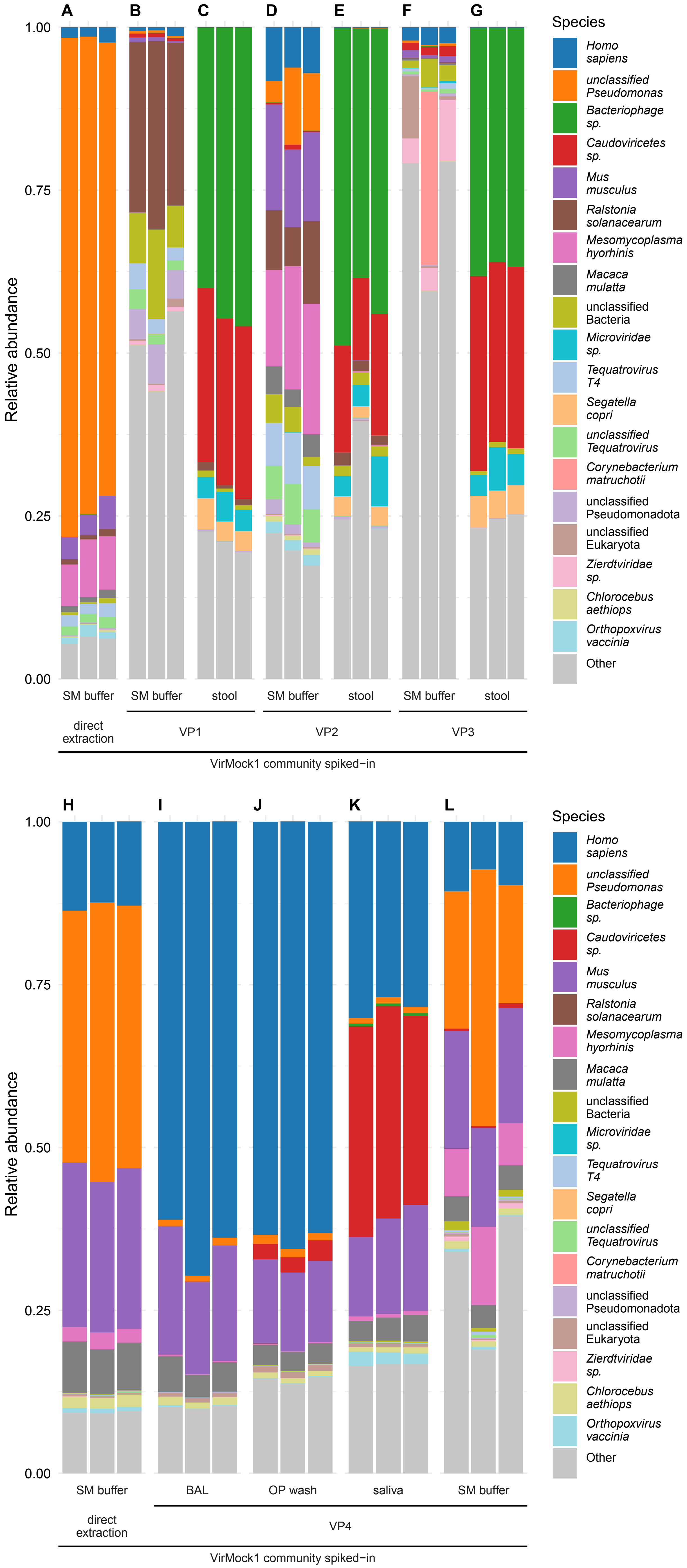
A
B
C
D
E
F
G
1.00
0.75
0.50
0.25
0.00
Relative abundance
SM buffer
SM buffer
stool
SM buffer
stool
SM buffer
stool
direct extraction
VP1
VP2
VP3
VirMock1 community spiked−in
Species
Homo sapiens
unclassified Pseudomonas
Bacteriophage sp.
Caudoviricetes sp.
Mus musculus
Ralstonia solanacearum
Mesomycoplasma hyorhinis
Macaca mulatta
unclassified Bacteria
Microviridae sp.
Tequatrovirus T4
Segatella copri
unclassified Tequatrovirus
Corynebacterium matruchotii
unclassified Pseudomonadota
unclassified Eukaryota
Zierdtviridae sp.
Chlorocebus aethiops
Orthopoxvirus vaccinia
Other
H
I
J
K
L
SM buffer
BAL
OP wash
saliva
SM buffer
direct extraction
VP4
VirMock1 community spiked−in

Figure S7

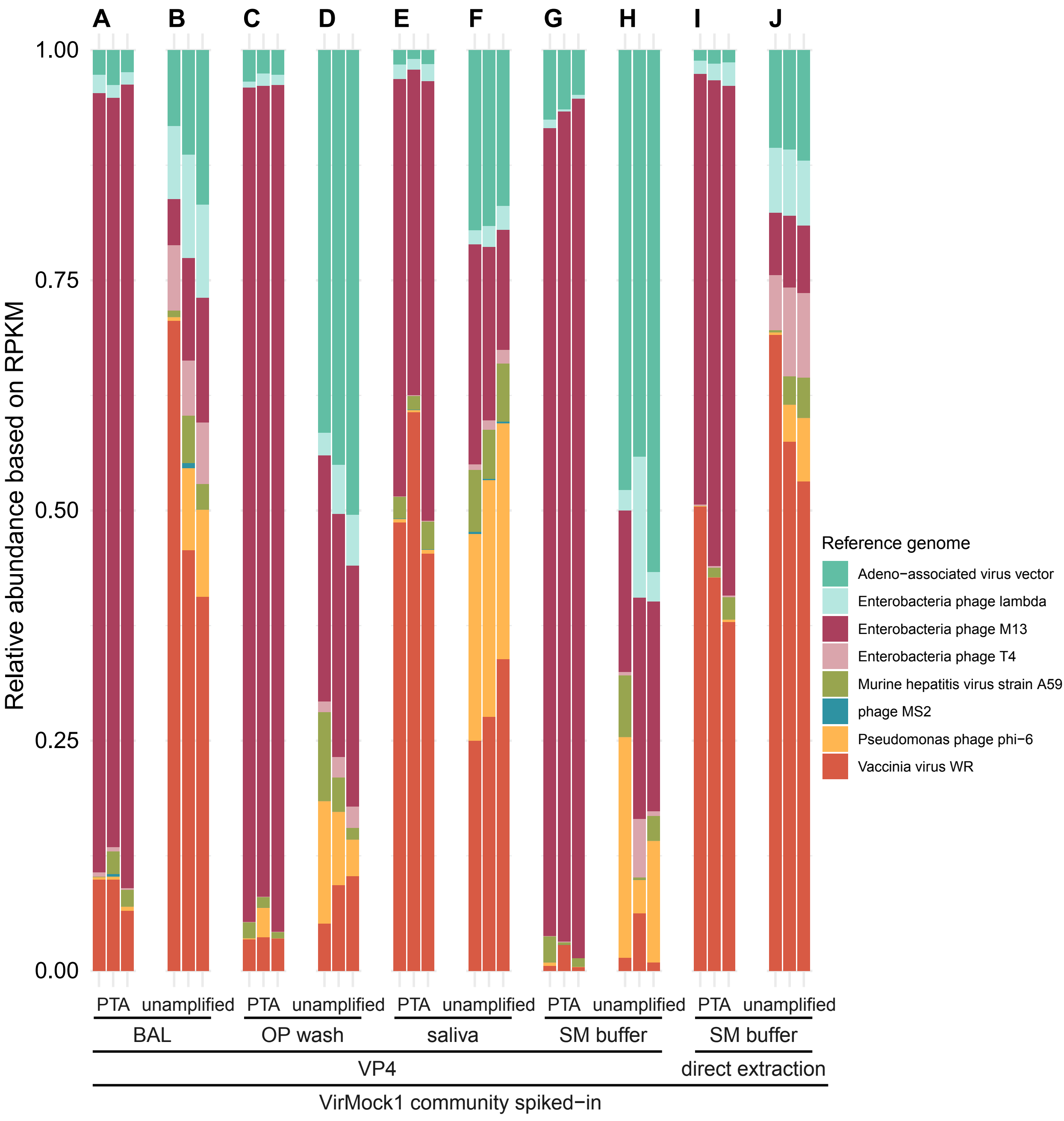

A
B
C
D
E
F
G
H
I
J
1.00
0.75
0.50
0.25
0.00
Relative abundance based on RPKM
PTA
unamplified
BAL
OP wash
saliva
SM buffer
SM buffer
VP4
direct extraction
VirMock1 community spiked−in
Reference genome
Adeno−associated virus vector
Enterobacteria phage lambda
Enterobacteria phage M13
Enterobacteria phage T4
Murine hepatitis virus strain A59
phage MS2
Pseudomonas phage phi−6
Vaccinia virus WR

Figure S8

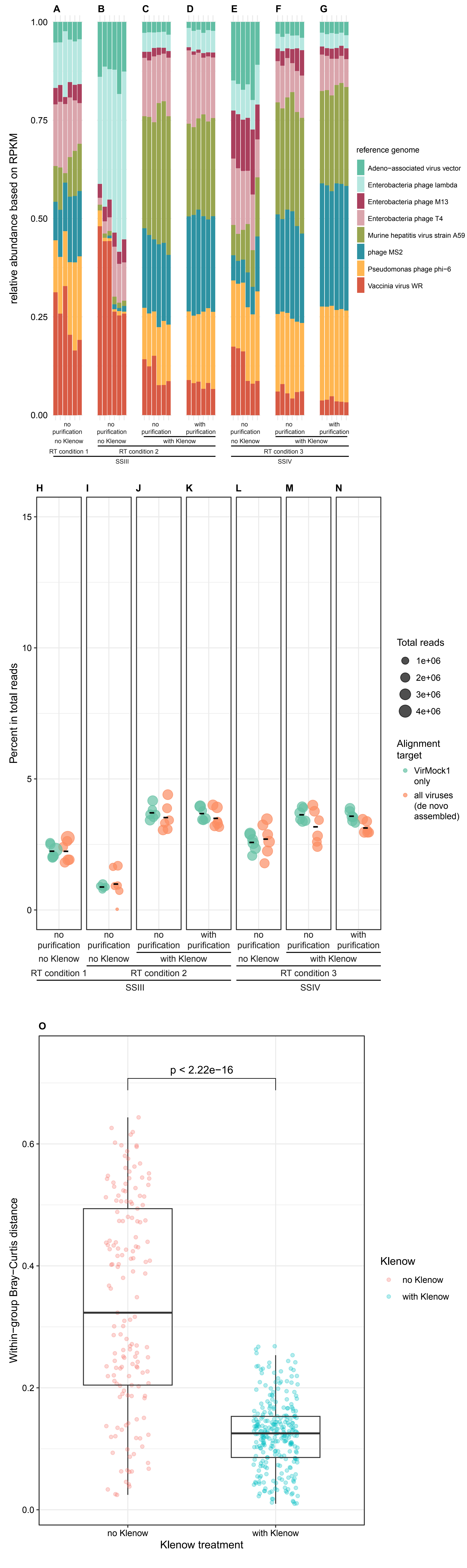
A
B
C
D
E
F
G
relative abundance based on RPKM
1.00
0.75
0.50
0.25
0.00
reference genome
Adeno−associated virus vector
Enterobacteria phage lambda
Enterobacteria phage M13
Enterobacteria phage T4
Murine hepatitis virus strain A59
phage MS2
Pseudomonas phage phi−6
Vaccinia virus WR
no purification
no purification
no purification
with purification
no purification
no purification
with purification
no Klenow
no Klenow
with Klenow
no Klenow
with Klenow
RT condition 1
RT condition 2
RT condition 3
SSIII
SSIV
H
I
J
K
L
M
N
Percent in total reads
15
10
5
0
Total reads
1e+06
2e+06
3e+06
4e+06
Alignment target
VirMock1 only
all viruses (de novo assembled)
no purification
no purification
no purification
with purification
no purification
no purification
with purification
no Klenow
no Klenow
with Klenow
no Klenow
with Klenow
RT condition 1
RT condition 2
RT condition 3
SSIII
SSIV
O
p < 2.22e−16
Within−group Bray−Curtis distance
0.6
0.4
0.2
0.0
Klenow
no Klenow
with Klenow
no Klenow
with Klenow
Klenow treatment

Figure S9

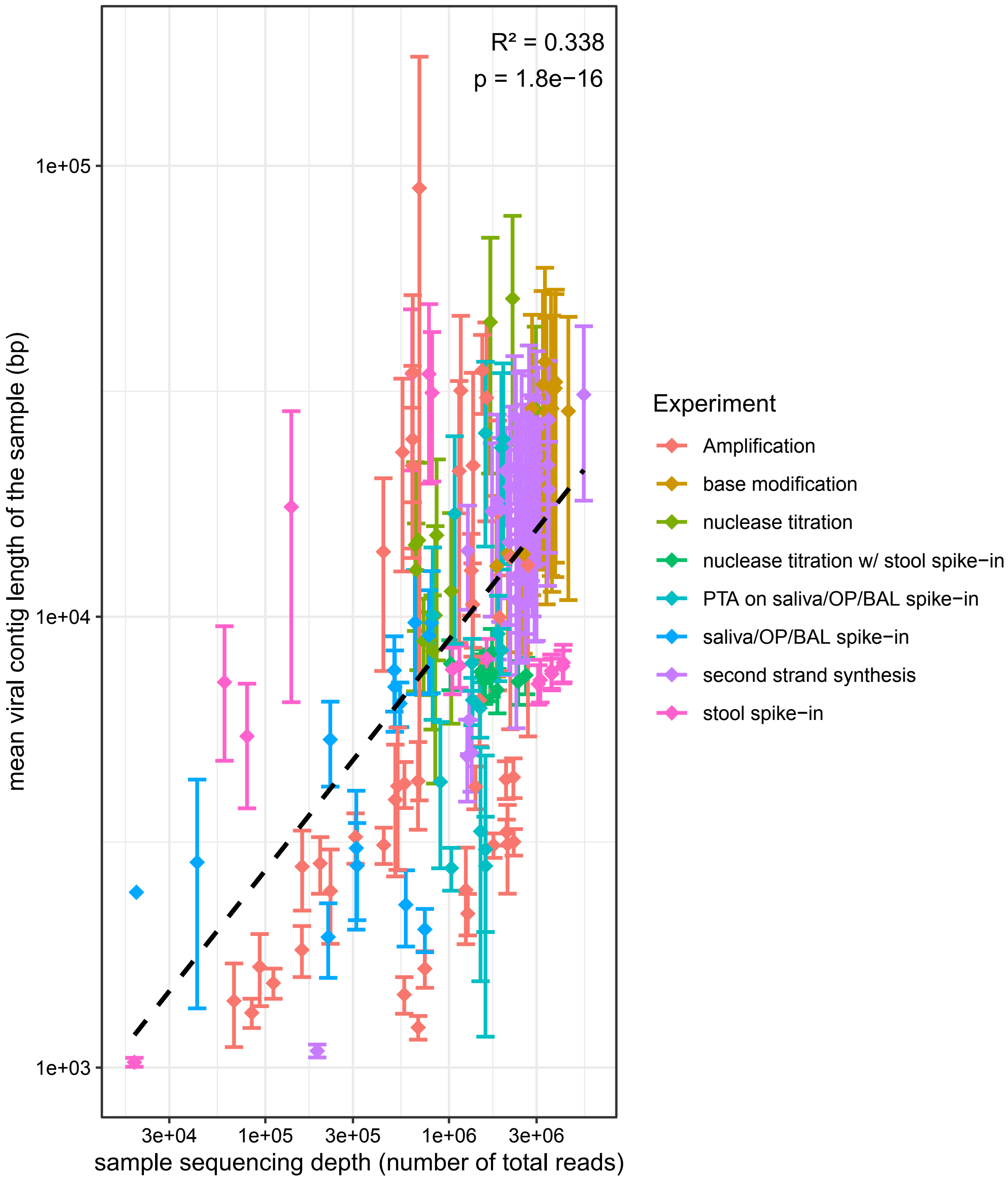
R² = 0.338
p = 1.8e−16
1e+05
1e+04
1e+03
mean viral contig length of the sample (bp)
3e+04
1e+05
3e+05
1e+06
3e+06
sample sequencing depth (number of total reads)
Experiment
Amplification
base modification
nuclease titration
nuclease titration w/ stool spike−in
PTA on saliva/OP/BAL spike−in
saliva/OP/BAL spike−in
second strand synthesis
stool spike−in

Figure S10

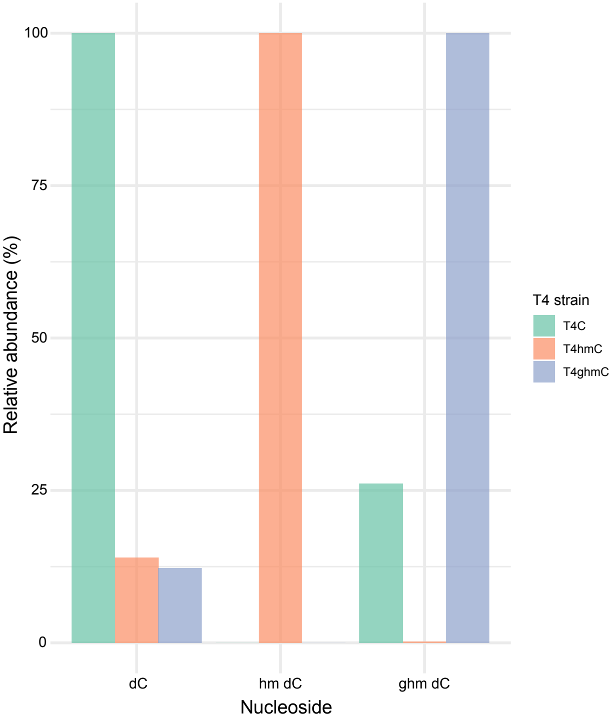
100
75
50
25
0
Relative abundance (%)
dC
hm dC
ghm dC
Nucleoside
T4 strain
T4C
T4hmC
T4ghmC

Figure S11

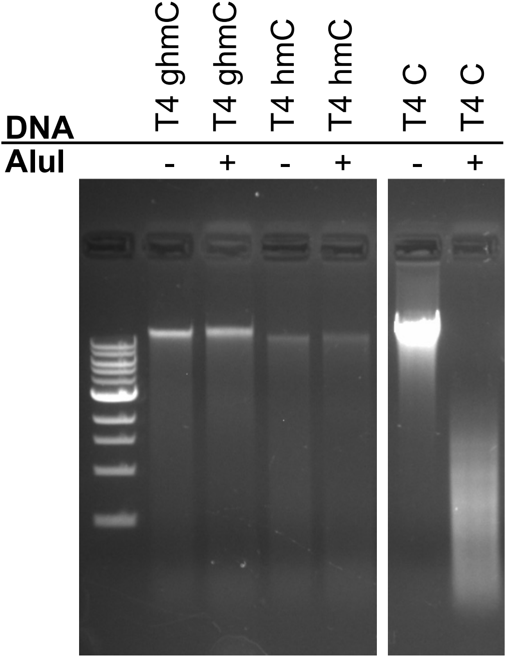

Supplement: Supplemental Figures — Fig. S1 to S11. [file msystems.00188-26-s0004.pdf]
